# Automated, multiparametric monitoring of respiratory biomarkers and vital signs in clinical and home settings for COVID-19 patients

**DOI:** 10.1073/pnas.2026610118

**Published:** 2021-04-23

**Authors:** Xiaoyue Ni, Wei Ouyang, Hyoyoung Jeong, Jin-Tae Kim, Andreas Tzaveils, Ali Mirzazadeh, Changsheng Wu, Jong Yoon Lee, Matthew Keller, Chaithanya K. Mummidisetty, Manish Patel, Nicholas Shawen, Joy Huang, Hope Chen, Sowmya Ravi, Jan-Kai Chang, KunHyuck Lee, Yixin Wu, Ferrona Lie, Youn J. Kang, Jong Uk Kim, Leonardo P. Chamorro, Anthony R. Banks, Ankit Bharat, Arun Jayaraman, Shuai Xu, John A. Rogers

**Affiliations:** ^a^Querrey Simpson Institute for Bioelectronics, Northwestern University, Evanston, IL 60208;; ^b^Department of Mechanical Engineering and Materials Science, Duke University, Durham, NC 27708;; ^c^Department of Biomedical Engineering, Northwestern University, Evanston, IL 60208;; ^d^Medical Scientist Training Program, Feinberg School of Medicine, Northwestern University, Chicago, IL 60611;; ^e^College of Computing, Georgia Institute of Technology, Atlanta, GA 30332;; ^f^Sibel Inc., Niles, IL 60714;; ^g^Sonica Health, Niles, IL 60714;; ^h^Max Nader Lab for Rehabilitation Technologies and Outcomes Research, Center for Bionic Medicine, Shirley Ryan AbilityLab, Chicago, IL 60611;; ^i^College of Medicine, University of Illinois at Chicago, Chicago, IL 60612;; ^j^Feinberg School of Medicine, Northwestern University, Chicago, IL 60611;; ^k^Division of Thoracic Surgery, Feinberg School of Medicine, Northwestern University, Chicago, IL 60611;; ^l^Wearifi Inc., Evanston, IL 60201;; ^m^Department of Materials Science and Engineering, Northwestern University, Evanston, IL 60208;; ^n^School of Chemical Engineering, Sungkyunkwan University, Suwon, 16419, Republic of Korea;; ^o^Department of Mechanical Science and Engineering, University of Illinois at Urbana–Champaign, Champaign, IL 61801;; ^p^Department of Surgery, Feinberg School of Medicine, Northwestern University, Chicago, IL 60611;; ^q^Department of Dermatology, Feinberg School of Medicine, Northwestern University, Chicago, IL 60611;; ^r^Department of Mechanical Engineering, Northwestern University, Evanston, IL 60208;; ^s^Department of Chemistry, Northwestern University, Evanston, IL 60208;; ^t^Department of Electrical Engineering and Computer Science, Northwestern University, Evanston, IL 60208;; ^u^Department of Neurological Surgery, Northwestern University, Evanston, IL 60208

**Keywords:** wearable electronics, digital health, biomarkers, respiratory disease, COVID-19

## Abstract

Capabilities in continuous monitoring of key physiological parameters of disease have never been more important than in the context of the global COVID-19 pandemic. Soft, skin-mounted electronics that incorporate high-bandwidth, miniaturized motion sensors enable digital, wireless measurements of mechanoacoustic (MA) signatures of both core vital signs (heart rate, respiratory rate, and temperature) and underexplored biomarkers (coughing count) with high fidelity and immunity to ambient noises. This paper summarizes an effort that integrates such MA sensors with a cloud data infrastructure and a set of analytics approaches based on digital filtering and convolutional neural networks for monitoring of COVID-19 infections in sick and healthy individuals in the hospital and the home. Unique features are in quantitative measurements of coughing and other vocal events, as indicators of both disease and infectiousness. Systematic imaging studies demonstrate correlations between the time and intensity of coughing, speaking, and laughing and the total droplet production, as an approximate indicator of the probability for disease spread. The sensors, deployed on COVID-19 patients along with healthy controls in both inpatient and home settings, record coughing frequency and intensity continuously, along with a collection of other biometrics. The results indicate a decaying trend of coughing frequency and intensity through the course of disease recovery, but with wide variations across patient populations. The methodology creates opportunities to study patterns in biometrics across individuals and among different demographic groups.

## Significance

Continuous measurements of health status can be used to guide the care of patients and to manage the spread of infectious diseases. Conventional monitoring systems cannot be deployed outside of hospital settings, and existing wearables cannot capture key respiratory biomarkers. This paper describes an automated wireless device and a data analysis approach that overcome these limitations, tailored for COVID-19 patients, frontline health care workers, and others at high risk. Vital signs and respiratory activity such as cough can reveal early signs of infection and quantitate responses to therapeutics. Long-term trials on COVID-19 patients in clinical and home settings demonstrate the translational value of this technology.

As of December 26, The Centers for Disease Control and Prevention (CDC) tabulations indicate over 18 million recorded cases of COVID-19 and more than 329,592 in deaths in the United States (1). Accurate and widespread testing is a key component of the response to this pandemic (2). Although the capacity and availability of COVID-19 molecular diagnostics continues to increase, shortcomings follow from variabilities in the accuracy of the tests, constraints in materials and supplies, long turnaround times associated with certain tests, inadequate access to testing sites, and a lack of human resources (3). An additional challenge is in limited prognostic tools to assess the trajectory of infection and the eventual need for hospitalization or mechanical ventilation. The CDC confirms that COVID-19 can be contracted via airborne transmission along with contact and droplet transmission—features that underscore the need to improve capabilities in risk stratification of exposures via contact tracing and to ensure sufficient quarantining for recovering individuals.

To address some of these needs, a range of digital health tools, from mobile applications for collecting self-reported symptoms to consumer wearable devices and clinical-grade medical sensors for tracking physiological status, are under development and in initial stages of deployment (4). Researchers at FitBit report the ability to identify infection with COVID-19 via four previous days of data collected from their wrist-worn devices to yield overnight heart rate, respiratory rate, and heart rate variability (5). Others claim similar detection capabilities with alternative wrist-based devices (6). Several ongoing large-scale trials aim to evaluate these wearables for early detection of COVID-19 infection, from smart rings (Oura Ring) to skin-interfaced patches [VitalConnect (7), Philips (8), Sonica (9)], to other smart watches [e.g., Empatica (10)] with support from various federal agencies. Devices that mount on the finger or wrist can monitor some subset of conventional vital signs (11–15), such as heart rate. Loose interfaces at these body locations, however, limit the range of detectable physiological activities, particularly respiratory signals (16, 17). The inability to capture complex health information reduces the potential for precise and reliable analysis (18). Development of robust metrics for early detection and disease tracking requires multiparametric operation across different digital biomarkers and unconventional metrics relevant to the disease of interest. Challenges remain in addressing these requirements simultaneously while maintaining simplicity and ease of use of the sensing system, as is necessary for practical deployment at scale in remote, continuous monitoring settings (19).

As COVID-19 is a respiratory disease, cough and other sounds from the thoracic cavity, trachea, and esophagus are examples of highly relevant biometrics. Laboratory-scale studies demonstrate cough-based diagnoses of diverse respiratory diseases through measurements of frequency (20), intensity (21), persistency (22), and unique audio features (23). Investigations on audio recording data show differences between COVID-19 positive and negative subjects’ vocalizing patterns including phonation of speech (24, 25), breathing, and coughing sounds (26–29). The results may suggest possibilities for disease monitoring in asymptomatic patients. Recent work applies voice profiling and computer audition to track cough, speech, respiratory, and other sounds for risk assessment and diagnosis of COVID-19 (30, 31). Monitoring cough and other vocal events (speaking, laughing, etc.) not only provides a signature of disease but also has potential in generating metrics of infectiousness, as these mechanisms yield aerosols/droplets that contribute to virus transmission (32–34). Previous studies show that the total volume of aerosols correlate with the loudness and duration of vocal events. Measurements of the timing and intensity of sounds may, therefore, serve as reliable means to quantify one aspect associated with risks of spreading the disease (35).

Point-of-care or semicontinuous methods for quantifying coughing or other vocal activities rely on electromyography, respiratory inductive plethysmography, accelerometry, or auditory recordings captured with one or several sensors, sometimes with other exploratory approaches (e.g., the nasal thermistor or the electrocardiography) (36–41). Digital signal processing followed by machine learning algorithms often serves as the basis for classification (42–53). Microphone-based methods prevail due to their widespread availability and their alignment with large crowd-sourced datasets (e.g., COUGHVID, HealthMode, DetectNow, VoiceMed). A key challenge is that background sounds and/or environmental noises frustrate robust and accurate measurements. Measurements of loudness can be unreliable because they depend on the separation between the device and the subject. Most importantly, audio recordings raise privacy and legal issues, thereby limiting the scale of application.

The results presented here bypass these disadvantages, to allow continuous assessments of respiratory biomarkers correlative to health status and droplet/aerosol production, with additional information on a range of traditional vital signs. Here, a simple, wireless monitoring device (54) combines with a cloud interface and a data analytics approach to allow continuous monitoring of a breadth of conventional (e.g., heart rate, respiratory rate, physical activity, body orientation, and temperature) and unconventional (e.g., coughing, speaking) physiological parameters of direct relevance to COVID-19. The results serve as a quantitative basis for 1) detecting early signs of symptoms in health care workers and other high-risk populations, 2) monitoring symptomatic progression of infected individuals, and 3) tracking responses to therapeutics in clinical settings. In addition, systematic studies presented here indicate that coughing, speaking, and laughing events measured with these devices correlate to the total amount of droplet production. This link offers an opportunity to quantify the infectiousness of individuals, as critical information in caring for patients and for improved risk stratification in the context of contact tracing and individual quarantines.

Pilot studies on COVID-19 patients at an academic medical center (Northwestern Memorial Hospital) and a rehabilitation hospital (Shirley Ryan AbilityLab) include 3,111 h of data spanning a total of 363 d from 37 patients (20 females, 17 males), in an overall implementation that supports automated operation, with minimal user burden. Long-term monitoring reveals trends in various parameters, including coughing frequency, following the test-positive date for eight patients (four females, four males) over more than 7 d. Evaluations across 27 patients (15 females, 12 males) with ages between 21 and 75 y reveal diverse coughing patterns across individuals and consistent trends during the recovery process.

## Results

### Sensor Designs, System Configurations, and Wireless, Cloud-Enabled Modes of Operation.

**Fig. 1.**
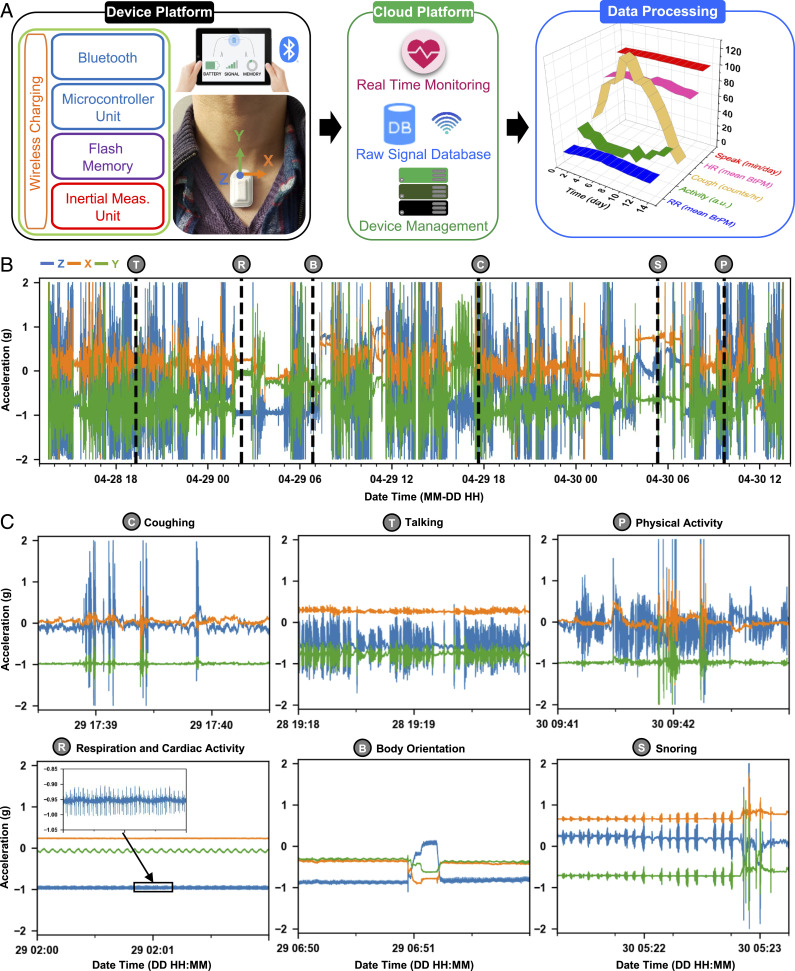
The health monitoring system incorporating an MA sensor, Bluetooth and cloud-based data transmission, automated data processing platform, and a user interface with a minimum need for manual operation. (*A*) Schematic illustration of the operational flow of the system, which consists of a device, cloud, and data processing platforms. (*B*) Sample three-axis acceleration raw data acquired continuously over 48 h on a COVID-19 patient. Dashed lines indicate occurrences of various representative body processes of interest, shown in (*C*) zoomed-in 2-min windows.

Fig. 1*A* presents a schematic illustration of the system. The circuit architecture represents an advanced version of the soft, skin-interfaced mechanoacoustic (MA) device reported previously (54). Briefly, a flexible printed circuit board (fPCB; 25-μm-thick middle polyimide with double-sided 12-μm-thick rolled, annealed copper, AP7164R, DuPont) with serpentine conductive traces supports collections of chip-scale components including a high-bandwidth, inertial measurement unit (IMU) with a triaxial accelerometer (LSMDSL, STMicroelectronics) as the key sensing element, a Bluetooth Low Energy (BLE) system-on-a-chip (SoC) for control and wireless connectivity, an on-board memory module for data storage, and a wireless unit for recharging a compact battery. A thin, soft elastomer membrane (Ecoflex, 00-30, smooth on, 300 μm) completely encapsulates the device as a compliant, nonirritating interface to the suprasternal notch (SN), supported by a thin, double-sided biomedical adhesive. The design of the system for the studies reported here includes an automated user interface that minimizes manual operations, where the wireless charging platform serves as a hub to switch modes from recording to data transfer. Specifically, the device remains in data acquisition mode when not on the charger. During charging, the device automatically stops recording and starts transmitting data to a BLE-enabled device such as a phone or a tablet with internet connectivity to a Health Insurance Portability and Accountability Act (HIPPA) compliant cloud server. Algorithms operating on the server deliver results to a graphical dashboard for feedback to health workers and/or patients.

When interfaced to the SN, the device captures subtle vibrations of the skin as signatures of a wide range of physiological processes (54). Fig. 1*B* shows an example of three-axis acceleration data recorded from an inpatient (female, age 53 y) wearing the device for 48 h. The sampling rate for motions perpendicular to the surface of the skin (*z* axis) is 1,666 Hz; the rates for the *x* axis (perpendicular to the axis of the neck) and *y* axis (along the neck) are 416 Hz. Fig. 1*C* shows time series representations of sample events in 2-min windows. Features associated with coughing and speaking include high-frequency components with significant amplitudes ($∼10^{○}\;g$) along the *z* and *y* axis but small amplitudes ($∼10^{-1}\;g$) along the *x* axis. Physical activity induces comparatively large accelerations ($∼10^{○}\;g$) along all axes. During the periods without such activities, subtle vital signals from respiratory and cardiac cycles are readily apparent. Recordings during sleep can also yield body orientations and snoring events, including those that are scarcely audible.

### Algorithm Development.

**Fig. 2.**
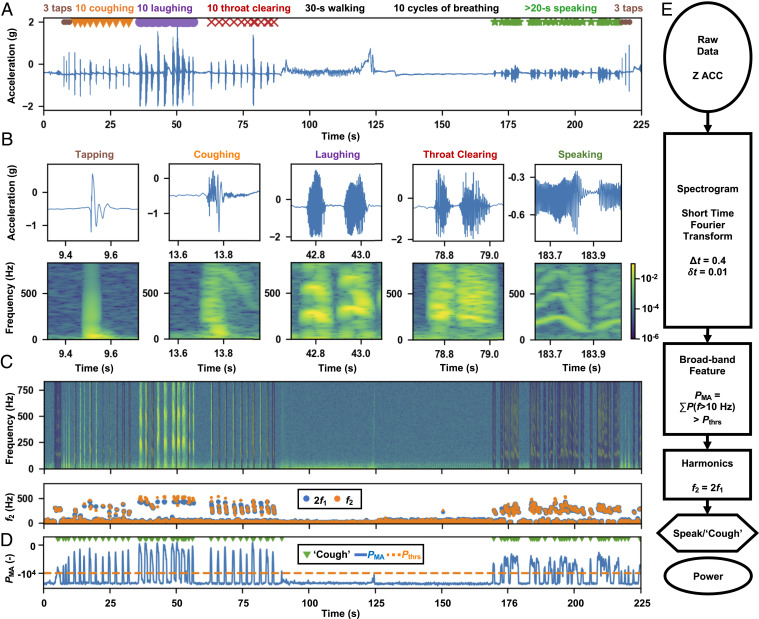
The signal preprocessing steps that identify broadband events of interest from quiet and speaking times from MA measurements. (*A*) The raw *z* axis data generated from controlled experiments on healthy normal subjects, with all of the events of interest repeated in sequence following a designed protocol (see *Materials and Methods* for details). (*B*) Example 400-ms clips of the raw *z* axis data and their corresponding spectrogram features. (*C*) Speaking signals distinct with a clear presence of harmonics ($P\left(f_{1}\right)$ and $P\left(f_{2}\right)$ of fundamental frequencies $f_{1}$ in the spectrogram analysis P(f), where $2f_{1}\approx f2$; see ref. 54 for details). Detected speaking periods are shaded in blue in the spectrogram. (*D*) After excluding speaking time, the detection of the high-frequency (f>10 Hz) MA power peaks with a minimum time interval of 0.4 s and a threshold of −10,000 yields time stamps for cough-like events that feature the impulse-like broadband acoustics. (*E*) A flow diagram summarizing the preprocessing steps that take in the raw *z* axis data and output the time stamps for cough-like and speaking events, along with their MA power, $P_{MA}$.

The focus here is on extraction of different vocal and respiratory events from these raw data. Methods for determining other important parameters, such as overall activity levels, heart rate, and respiration rate, can be found elsewhere (54). In the context of COVID-19, a particular interest is in identifying and tracking coughing events, in the presence of other MA signals. Fig. 2 presents a scheme for data preprocessing that exploits time–frequency features to differentiate coughing from other common daily activities. Algorithm development uses recordings captured from 10 healthy normal subjects in controlled experiments with a protocol (see *Materials and Methods* for details) that generates a large number of events of interest in various body postures. Fig. 2*A* shows typical *z* axis data from a representative experimental session. Each testing sequence begins and ends with three taps of the fingers on the device as time stamp markers. In between are consecutive 10 forced coughs, 10 laughing events, 10 throat clearing events, 30 s of walking, 10 cycles of breathing, and more than 20 s of speaking. Fig. 2*B* shows time series and spectrogram representations of such events, the latter of which uses short-time Fourier transform and a Hanning window with a width Δt = 0.4 s moving in time steps of δt = 0.01 s. The algorithm considers each set of windowed data independently in the process of cough determination. The coughing signals feature a broad-bandwidth impulse-like response, followed usually by a high-frequency chirp (>200 Hz). Speaking signals also have high-frequency components, but usually with distinct harmonic features. An algorithm based on such harmonics can screen the data for prominent speaking periods (Fig. 2*C*). After excluding speaking events, a minimum amplitude threshold $P_{thrs}$ = −10,000 detects peaks of the logarithm of spectral power integrated across the high-frequency band (>10 Hz) ($P_{MA}$) and labels them as cough-like events, with a minimum time interval between peak events of 0.4 s (Fig. 2*D*). Here, cough-like events include laughing, throat clearing, and also some speaking periods that exhibit unclear harmonics. Fig. 2*E* shows the data processing flow, which begins with raw *z* axis data and returns the time stamps for speaking and cough-like events, as well as their associated integrated logarithm power. Such an analysis applied to the testing data detects 26.4 s of speaking with clear harmonics features, and identifies 10 coughing, 20 laughing, 12 throat clearing, 36 speaking, and 6 tapping instances as cough-like (Fig. 2*A*).

**Fig. 3.**
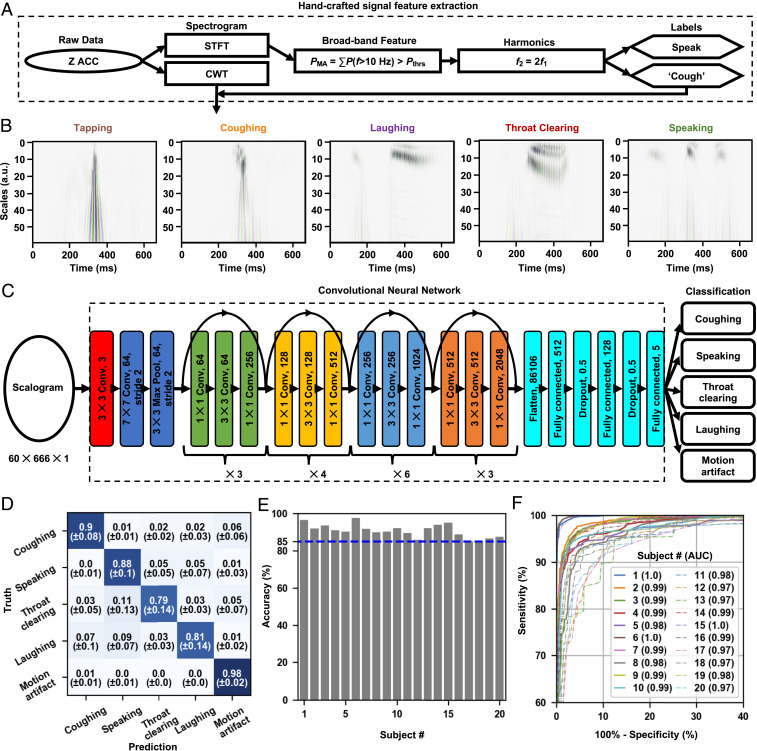
The machine learning algorithm for the classification of cough-like events extracted by the preprocessing algorithm. (*A*) Steps of feature scalogram generation from raw data. (*B*) Representative scalograms of events of interest. (*C*) The architecture of a CNN that takes in a feature scalogram and outputs its probabilities of classes. (*D*) The averaged confusion matrix from the iterated 20 leave-one-out testings. (*E*) The overall testing accuracy on each left-out subject using a model trained on the other 19 subjects. (*F*) The macroaveraged ROC curves of each left-out subject using a model trained on the other 19 subjects and the corresponding AUC. a.u., arbitrary unit.

Distinguishing actual coughs from the pool of cough-like events demands further classification by machine learning. A convolutional neural network (CNN) uses as inputs Morlet wavelet transforms of 0.4-s raw *z* axis data (shaped by the Hanning window) of these events (Fig. 3*A*). The wavelet transform offers advantages compared to the short-time Fourier transform because of its favorable resolution in characterizing nonstationary signals, which improves the accuracy of classification. Fig. 3*B* shows scalograms of cough-like events, including tapping (one type of motion artifact), coughing, laughing, throat clearing, and speaking events. These scalograms, with shapes of 60×666×1, serve as inputs to the CNN model. As shown in Fig. 3*C*, the CNN starts with a three-channel convolutional layer with a kernel size of 3×3, followed by a standard 50-layer residual neural network (ResNet), a CNN architecture for image classification (55). The output of the ResNet flattens to a layer of 86,106 neurons, followed by two fully connected layers with rectified linear unit activation and two dropout layers (p=0.5) alternately. The final fully connected layer of the CNN model has five neurons with Softmax activation, which corresponds to probabilities associated with the five types of events of interest: coughing, speaking, throat clearing, laughing, and motion artifact, where most of the motion artifacts are those events arising from physical contact on or around the device.

Data collected from 10 healthy volunteers yield labeled time windows consisting of 1,379 coughing, 1,441 speaking, 1,313 laughing, 1,423 throat clearing, and 2,890 motion artifact events. Because sample events generated in controlled experiments can differ from those that occur naturally in uncontrolled settings, the training of the CNN model uses not only scalograms of labeled events from 10 healthy volunteers (subjects 1 to 10) but also 10 COVID-19 patients during natural daily behaviors (subjects 11 to 20). Determinations of ground truth from the patient data involve listening to soundtracks created from the accelerometer data and then manually labeling the data (see *Materials and Methods* for code availability). Most of the events associated with coughing, speaking, and motion artifacts can be determined unambiguously in this manner. Difficulties arise in distinguishing between laughing, throat clearing, and certain periods of speaking, thereby leading to some level of uncertainty. Such manual analysis of data collected from 10 COVID-19 patients generates a total of 1,405 coughing, 1,449 speaking, 193 laughing, 210 throat clearing, and 2,905 motion artifact events. *SI Appendix*, Table S1 includes detailed demographic and data collection information for all of the training subjects.

The generalization performance of the CNN model can be determined using a leave-one-out strategy, where one leaves a subject out of the training set (19 subjects for training) and then tests the trained model on this subject. Iterations apply this approach to each of the 20 subjects. Each training set consists of a random collection of 80% of the labeled events from the 19 subjects, with the remaining 20% used for validation. The training uses an Adam optimization algorithm. Fig. 3*D* shows the averaged confusion matrix of 20 leave-one-out testing cycles. The model achieves accuracies of 0.90±0.08 for coughing, 0.88±0.1 for speaking, 0.79±0.14 for throat clearing, 0.81±0.14 for laughing, and 0.98±0.02 for motion artifact. The classifications for throat clearing and laughing have comparatively lower average accuracies and higher standard deviations, due to their similarity to certain speaking signals, as evidenced by the confusion matrix (Fig. 3*D*). Fig. 3*E* shows the overall five-way classification accuracies on each subject using a model trained on the other 19 subjects. The minimum overall accuracy is 0.85 for all subjects. The receiver operation characteristic (ROC) curve characterizes the trade-off between sensitivity and specificity in binary classification—varying the threshold of the cutoff probability at the final output layer generates ROC curves of each of the five types of events (coughing vs. noncoughing, speaking vs. nonspeaking, etc.). Fig. 3*F* presents the macroaveraged ROC curves for each subject. The high area under the curve (AUC) of >0.97 for all subjects indicates that the model achieves a good balance between sensitivity and specificity (see *SI Appendix*, Table S2 for detailed information).

### MA Sensing of Droplet Production.

**Fig. 4.**
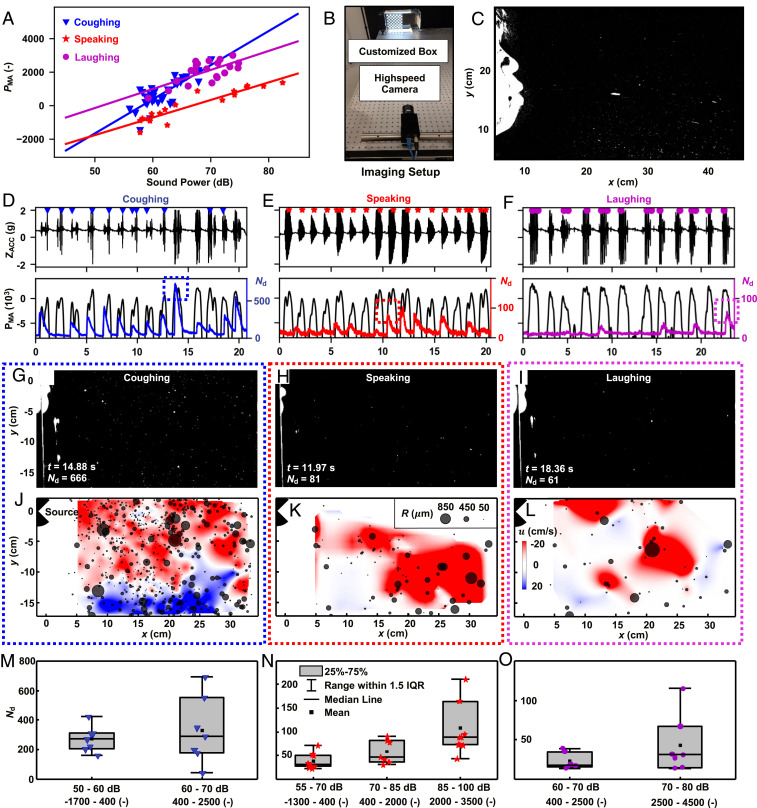
MA sensing to quantify the transmission of droplets. (*A*) MA power vs. decibel meter measurement for coughing, speaking, and laughing. (*B*) Experimental setup for optical imaging of droplets. (*C*) Sample image of coughing. (*D*–*F*) Time series of MA *z* axis acceleration (Z_ACC_) in sync with the analysis of MA power and the imaging detection of the number of the particles. (*G*–*I*) Instantaneous images of coughing, talking, and laughing at the peak of corresponding marked boxes in *D*–*F*. (*J*–*L*) Detected particles with sizes indicated by the diameters of the gray circular symbols, overlapped with velocity contour fields at the corresponding instances in *G*–*I*; the color denotes stream-wise velocity in the horizontal (*x* axis) direction. (*M*–*O*) Box and whisker plots showing the number of particles with mean, median, and interquartile range (IQR) for all measured cycles of coughing, speaking, and laughing, respectively. See *Materials and Methods* for full description.

Given the transmissibility of many types of viruses through droplets and aerosols, MA measurements that correlate the timing and intensity of activities associated with droplet production may yield reliable metrics of the risks of the population spread of COVID-19. Robust identification of coughing events, along with their frequency, intensity, and, in the future, detailed time dynamics (i.e., effective sounds), has relevance in this context. Other forms of vocalization such as speaking, singing, shouting, etc., are also important. Previous studies show that different types and volumes of vocal or respiratory-related events yield significantly different levels of aerosol production (35), with direct relevance to evaluating the risks of viral transmission. Fig. 4*A* presents results that calibrate the high-frequency power $P_{MA}$ associated with the *z* axis acceleration component of the MA signals to measurements with a decibel meter $P_{dB}$ in a quiet (background noise of <40 dB) environment for cases of coughing, speaking (repeating words “terminator”), and laughing from a healthy normal subject (male, Asian, age 30 y). The results show a linear correlation $P_{MA}=p_{1}P_{dB}+p_{2}$ for all three classes in the audible range of 55 dB to 85 dB, with $p_{1}=200\pm 20$ dB^−1^, $p_{2}=-12,000\pm 1,700$ dB^−1^ for coughing; $p_{1}=105\pm 10$ dB^−1^, $p_{2}=-7,000\pm 700$ dB^−1^ for speaking; and $p_{1}=114\pm 30$ dB^−1^, $p_{2}=-5,800\pm 1,200$ dB^−1^ for laughing (*SI Appendix*, Fig. S1).

Fig. 4 *B* and *C* shows the experimental setup of quantitative imaging studies (see *Materials and Methods* for details) that examine correlations between MA data and droplet production, with a focus on relationships between the total number of droplets and the intensities of coughing, speaking, and laughing. The measurements include droplet dynamics captured via particle tracking velocimetry (PTV; see *Materials and Methods* for details), power levels from the MA data ($P_{MA}$), and audio levels from a decibel meter ($P_{dB}$). Fig. 4 *D*–*F* shows a sequence of results from the MA sensor and the PTV analysis for coughing, speaking, and laughing, respectively, where markers indicate events correctly identified and classified by the automated algorithm. Fig. 4 *G*–*I* are images of coughing, talking, and laughing at the peak of corresponding marked boxes in Fig. 4 *D*–*F*. The PTV method tracks individual particles in the Lagrangian frame of ref. 59. Fig. 4 *J*–*L* shows the detected particles, with sizes indicated by the diameters of the gray circular symbols. As expected, the findings indicate that a larger number of droplets (determined across the investigation area of ∼34×∼17 cm^2^, and with radius R > 50 μm in the detectable range) results from coughing (200 to 800 droplets) than from speaking or laughing (10 to 200 droplets) at comparable decibel levels and time durations. More than 60% of droplets are smaller than 150 μm in radius for all measured respiratory activities (*SI Appendix*, Fig. S2).

Interpolated horizontal velocity (u) contours from droplet trajectories indicate a large swirling motion for coughing, with positive velocity near the mouth and negative velocity in the bottom of the investigated area (Fig. 4*J*). Droplets show ballistic behavior for speaking and dispersive behavior for laughing (Fig. 4 *K* and *L*). The ballistic behavior of droplets results from enhanced jet-like transport of the expelled airflow induced by plosive sounds (56). Drastically different inertial particle dynamics occur depending on the size of droplets, even within the same cycle. Specifically, small droplets linger in the air and respond to ambient flows. Large droplets travel at high velocities and are minimally influenced by flows, within a range investigated. Statistical analyses of the total number of droplets ($N_{d}$) of all measured respiratory activities at various audio levels appear in Fig. 4 *M* and *O*. The number of droplets exhibits some correlation to the audio decibel level and the power intensity of the MA data, for all activities. *SI Appendix*, Fig. S3 and Movie S1 include additional results from the imaging analysis of droplet dynamics.

### Multiparametric Monitoring from a Cohort of COVID-19 Patients.

**Fig. 5.**
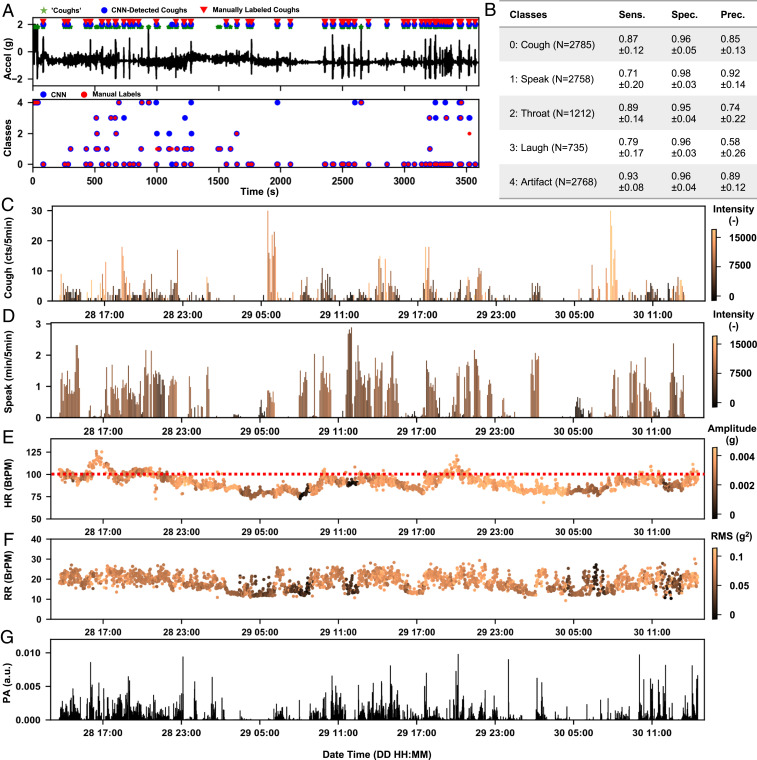
Deployment of MA devices on to the COVID-19 patients in clinical settings. (*A*) Representative *z* axis acceleration data measured from a female patient. The automated algorithm detects cough-like events and outputs five-way classification for the events to coughing (0), speaking (1), throat clearing (2), laughing (3), and motion artifacts (4). (*B*) The macroaveraged testing performance (sensitivity/recall, specificity, and precision) of each type of event on the 10 patients with manual labels, which include 10,258 randomly sampled events in total. (*C* and *D*) Example results for the detected coughing and talking frequency and intensity (color-coded) in 5-min windows from continuous 48-h monitoring of the same patient (raw acceleration data are shown in Fig. 1 *B* and *C*). (*E*–*G*) The vital signs information includes heart rate (HR) in a unit of beats per minute (BtPM) and respiration rate (RR) in a unit of breaths per minute (BrPM), and physical activity (PA), extracted from the same measurement, with their amplitude information color coded. a.u., arbitrary unit.

Scaled deployment of the MA device and the machine learning algorithm on COVID-19 patients in a clinical setting demonstrates practical utility and patient compliance without user or physician burden. The studies involve continuous, long-term (>7 d) monitoring of parameters relevant to patient status, not only coughing dynamics but also other forms of vocalization, along with heart rate, respiration rate, body orientation, and overall activity. These pilot studies correspond to 3,111 h of data from 37 patients (20 females, 17 males; see *SI Appendix* for detailed demographic information) with 27,651 detected coughs. Fig. 5*A* shows data and analysis results for a representative 1-h session with a female patient. The CNN model, trained using a process that is blind to any of the patients described in this section, returns predicted classes for each cough-like event detected by the preprocessing step. A manual labeling process based on audio files provides reference labels for comparison. Statistical analysis, on a total of 10,258 randomly sampled events from 10 patients (6 females, 4 males; patient IDs listed in *SI Appendix*, Table S1) with manual labels shows macroaveraged sensitivity (i.e. recall) of ≥0.87, specificity of ≥0.96, and ≥0.85 precision for coughing (*N* = 2,785) and artifacts detection (N=2,768) (Fig. 5*B* and *SI Appendix*, Table S2). The sensitivity and precision for speaking (N=2,758), throat clearing (N=1,212), and laughing (N=735) are as low as 0.58, likely due, in part, to the ambiguities in ground truth labeling. *SI Appendix*, Table S2 includes additional details on statistical analyses with subject-specific information. Fig. 5*C* presents results of coughing counts per 5 min in bars and the associated coughing effort (i.e., $P_{MA}$) in color. In general, the coughing frequency and intensity peak in the morning, and distribute evenly throughout the day. Fig. 5*D* presents a similar analysis of speaking, with uniformly distributed speaking time and loudness (i.e., $P_{MA}$) during daytime.

**Fig. 6.**
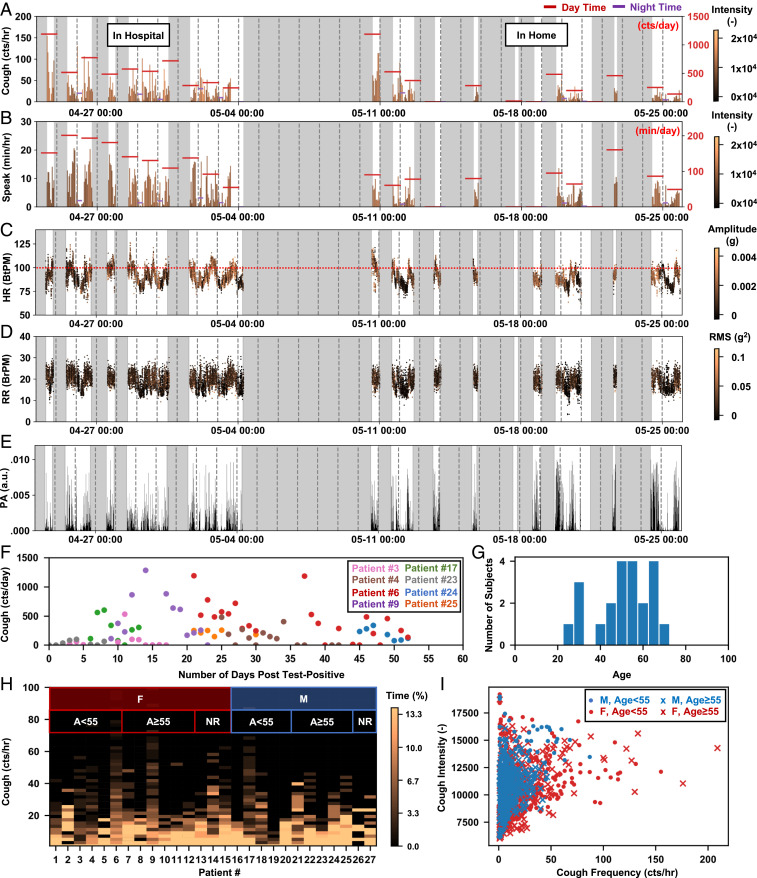
Long-term monitoring of coughing and other biometrics of COVID-19 patients. Long-term MA sensing of (*A*) cough frequency per hour, (*B*) talk time per hour, (*C*) heart rate, (*D*) respiration rate, and (*E*) physical activity for the same patient shown in Fig. 5 *A* and *C*–*G*, with the intensity or amplitude information of the associated events color coded in each time bin. (*F*) The time series plot of coughing counts organized in days post the test-positive date from eight COVID-19 patients. (*G*) The age distribution of the 27 patients whose data are not used to build the machine learning model. (*H*) The histogram of coughing frequency of the 27 patients. Ages for 3 females and 2 males are not reported (NR). (*I*) The cough intensity versus cough frequency analyzed for each hour of data, clustered by four demographic groups. a.u., arbitrary unit; BrPM, breaths per minute; BtPM, beats per minute.

Previously reported algorithms applied to these same MA data streams yield other important parameters (54). For example, Fig. 5 *E*–*G* summarizes heart rate, respiration rate, and physical activities, where the color-coded intensity values correspond to peak amplitudes of cardiac signals in the frequency band 20 Hz to 55 Hz and root-mean-square values for low-passed respiration cycles in the band 0.1 Hz to 1 Hz. Fig. 6 *A*–*E* presents this collective information (coughing counts, speaking time, heart rate, respiration rate, and physical activity, and their associated intensity or amplitude) for the same patient over 1 mo. Gray shaded areas indicate periods when the patient is not wearing the device. The same analysis has been applied to a total of 27 patients (15 females, 12 males) whose data are not used in building the CNN model. *SI Appendix*, Figs. S4–S20 shows the results for an additional 17 patients (9 females, 8 males; patient IDs listed in *SI Appendix*, Table S1) with a minimum of 7 d of enrollment.

Fig. 6*F* presents a time series plot for eight patients (four females, four males; patient IDs listed in *SI Appendix*, Table S1) with the date of a positive PCR test for COVID-19, where the event of interest is coughing count organized by days after the test. The results suggest a correlation between coughing frequency and the gradual process of recovery, as might be expected. The significant variation in decay rates, however, indicates individual-specific recovery and aerosolization potential. Fig. 6*G* summarizes the age distribution for the total of 27 testing patients. Fig. 6*H* compares the histogram of coughing frequency of these individuals, to reveal the diverse regularity of coughing across time. Fig. 6*I* shows the coughing frequency versus the average coughing intensity for all hourly measurements, clustered into four demographic groups (males of age <55 y, males of age ≥55 y, females of age <55 y, females of age ≥55 y). The available results suggest that females tend to cough more than males. *SI Appendix*, Table S1 includes detailed demographic and data collection information for all of the testing patients. The statistics may provide insights for creating guidelines for disease management and containment. Further studies on an expanded patient population with detailed demographic information are, however, necessary to enable big-data–based studies of the demographic dependence and/or individual variance of relevant biometrics.

## Discussion

This paper introduces an automated hardware–software solution for sensing of diverse health information relevant to patient status, with a focus on underexplored respiratory biomarkers such as cough and their changes with COVID-19 disease state. Scaled studies indicate applicability to COVID-19 patients in both clinical and home settings. The approach relies on a soft, wireless sensing device placed on the SN, to capture data that can be processed through a combination of digital filtering and machine learning techniques to separate and quantify different body processes. In addition to patient status, these data show promise in tracking droplet/aerosol production and, therefore, disease transmission related to cough and other expiratory events. The results have implications for early detection, patient care, and disease management, with specific relevance to COVID-19.

These systems allow for multiparametric monitoring with minimal burden, through a range of conventional and unconventional signatures of health status. Cough is an example of a potentially important biomarker that can yield insights to complement those from analysis of traditional vital signals. Extensions of the approaches reported here can be considered in strategies that extract additional information from specific forms of speech (e.g., plosive consonants), advanced assessments of coughing and respiratory sounds, and correlations between body positions and these activities, as well as coupled responses and timing intervals between different events. MA sensing of distinctive features in respiratory biomarkers and physiological characteristics between COVID-19 patients and healthy subjects suggests a versatile platform for disease monitoring and management. The addition of optical sensors will enable measurements of blood oxygenation, without affecting the ability to simultaneously capture MA signals. The results offer many possibilities in data fusion for precision healthcare, including but not constrained to COVID-19 (19, 57, 58). Scaled deployment will yield large amounts of accessible biometric data, as the potential basis for predictive disease models, cost-effective care of patients, and containment of disease transmission.

## Materials and Methods

### Device Design and Components.

The fPCB schematic diagram and board layout were designed using AUTODESK EAGLE (version 9.6.0) for a stretchable and bendable MA device. Serpentine-shaped outlines connect three separated islands (main body, sensor, and charging coil). A summary of the bill of materials for the device includes 0201 and 0402 inch footprint (imperial code) passive components (resistors, capacitors, and inductors), four turns of wireless charging coil pattern (resonance frequency: 13.56 MHz), full-bridge rectifier, power management integrated circuits (IC) (Bq25120a, Texas Instruments), 3.0-V step-down power converter (TPS62740, Texas Instruments), 3.7-V lithium polymer battery (75 mAh), voltage and current protection IC for Li-Polymer battery (BQ2970, Texas Instruments), BLE SoC (nRF52840, Nordic Semiconductor), flash memory (MT29F4G, Micron), and IMU (LSM6DSL, STMicroelectronics).

### Device Fabrication and Encapsulation.

Panels of fPCB were manufactured, and surface-mount device processes were performed by an International Organization for Standardization 9001-compliant manufacturer. Customized firmware was downloaded by Segger Embedded Studio, followed by an fPCB folding and battery soldering process. Each aluminum mold for top and bottom layers was prepared with a freeform prototyping machine (Roland MDX 540), and the devices were encapsulated using precured top and bottom layers (Silbione-4420, each 300 μm thick) after filling with silicone elastomer (Eco-Flex 0030, 1:1 ratio) in the cavity in which the device was positioned. After fixing and pressing top/bottom molds using clamps, the mold was placed into an oven that holds a temperature of 95 °C for 20 min to cure the silicone elastomer. The mold was then taken out of the oven and placed in a room temperature area for 20 min to cool down. After cooling down, the clamps were removed, the encapsulated device was placed on a cutting surface, and excess enclosure material was removed using a prefabricated hand-held die cutter. A CO_2_ laser formed the shape of the double-sided adhesives and yielded a smooth and clean contour cut.

### Data Collection.

All of the participants provided written/verbal consent prior to their participation in this research study (see *SI Appendix*, Table S1 for demographic information of all individuals studied). Study procedures were approved by the Northwestern University Institutional Review Board (STU00202449 and STU00212522) and were registered on ClinicalTrials.gov (NCT02865070 and NCT04393558). All study-related procedures were carried out in accordance with the standards listed in the Declaration of Helsinki, 1964. During the study, participants wore an MA device at SN (Fig. 1*A*). In the case of patients, a clinician/research staff assisted in placing the sensor.

Healthy controls were asked to perform 18 repetitions of the following sequence of activities with some variability in the intensity of each of the activities over a 2- to 4-h period: three taps on the sensor, 10 coughs, 10 laughs, 10 throat clearings, 30 s of walking, 10 cycles of breathing (inhale and exhale), more than 20 s of speaking, and three taps on the sensor. Of these repetitions, sedentary activities in five sets were performed while sitting, five sets during standing, and eight sets while lying down (two in supine, two in prone, two in left recumbent, and two in right recumbent) positions. In the case of patients, a reduced set of activities were used at the beginning of each test, which included three taps on the sensor, five coughs, five cycles of deep breathing, and three taps on the sensor.

### Sterilization Process.

After each use, the MA sensor was thoroughly disinfected/cleaned with isopropyl alcohol (70% or above) or Oxivir TB wipes (0.5% hydrogen peroxide) and left to dry at room temperature, and the same process was repeated twice.

### Convolutional Neural Network.

The CNN starts with a convolution with a kernel size of 3 × 3 and three different kernels, followed by a standard 50-layer ResNet as described in detail in ref. 55. At the output of the ResNet, a flattening layer of 86,106 neurons follows. Finally, three fully interconnected layers with 512, 128, and 5 neurons, respectively, and two dropout layers with *P* = 0.5 follow alternately. The CNN uses an Adam optimizer for training. The training process follows a leave-one-out strategy, where one leaves a subject out of the training set (19 remaining subjects for training) and then tests the trained model on this subject. Each training set applies a fivefold cross-validation procedure. This approach iterates through each of the 20 subjects. *SI Appendix*, Table S2 includes detailed information on the cross-validation results for each subject.

### Data Analytics.

All analysis used Python 3.0 with SciPy, PyWavelets, and TensorFlow packages.

### Code Availability.

The codes used for audio soundtrack conversion and manual labeling processes are available on GitHub at https://github.com/nixiaoyue/MA-cough. The analysis codes used in this study are available from the authors upon request.

### Droplet Dynamics via PTV.

Droplet dynamics of coughing, speaking, and laughing were quantified by PTV. Coughing, speaking (the word “terminator” was used), and laughing were repeated 14, 26, and 15 times, respectively, at various decibel levels. More data samples for speaking were collected to cover a wider range of decibels up to 100 dB. Each respiratory activity was performed in the customized box made of acrylic glass with an inner dimension of 45×30×30 cm^3^ (L × W × H). The investigation area for tracking droplets was ∼34×∼17 cm^2^ illuminated by 16 arrays for 600 lumen LED light bars. PTV experiments were recorded by a 2,048 × 1,088 Emergent HT-2000M with 50-mm F1.4 manual focus Kowa lens at the frame rate of 338 frames per second. To achieve continuous and simultaneous measurements with MA sensor and audio meter (Decibel X, calibrated by SD-4023 sound level meter and R8090 Sound Level Calibrator), approximately 10,000 frames were recorded for each respiratory activity. Preprocessing, calibration, tracking, and postprocessing are performed by a previously developed PTV code (59). Image sequences were preprocessed by subtracting the background noise and enhancing the contract. Droplets are detected at the subpixel level with the area estimation. The scattering cross-section of a detected droplet and refractive index of droplet as well as the surrounding medium, air, and wavelength of the light source were used to calculate the actual radius of detected droplets based on the Mie scattering theory (60, 61). The minimum radius of droplets measured in this work is ∼60 μm. Detected droplets were tracked using the Hungarian algorithm and linked by performing a five-frame gap closing to produce longer trajectories. Velocity and Lagrangian acceleration were filtered and computed using fourth-order B splines. Vector contour fields were obtained by interpolating scattered Lagrangian flow particles at each frame based on the natural neighbor interpolation method.

## Supplementary Material

Supplementary File

Supplementary File

## Data Availability

All relevant data are included in the article and *SI Appendix*. Additional supporting data are available from the corresponding authors on request. All request for raw and analyzed data and materials will be reviewed by the corresponding authors to verify whether the request is subject to any intellectual property or confidentiality obligations. Patient related data not included in the paper were generated as part of clinical trials and may be subject to patient confidentiality.
