# Supplementary material for: Automated, multiparametric monitoring of respiratory biomarkers and vital signs in clinical and home settings for COVID-19 patients
Source: Proc Natl Acad Sci U S A. 2021 Apr 23;118(19):e2026610118. doi: 10.1073/pnas.2026610118 (PMC8126790; doi:10.1073/pnas.2026610118)
Supplement: Supplementary File [file pnas.2026610118.sapp.pdf]

Supplementary Information for

Automated, multiparametric monitoring of respiratory biomarkers and vital signs in clinical and home settings for COVID-19 patients

Xiaoyue Ni, Wei Ouyang, Hyoyoung Jeong, *et al.*

Corresponding Author name.
E-mail: stevexu@northwestern.edu, jrogers@northwestern.edu

This PDF file includes:

Figs. S1 to S20 (not allowed for Brief Reports)
Tables S1 to S2 (not allowed for Brief Reports)
Legend for Movie M1

Other supplementary materials for this manuscript include the following:

Movie M1

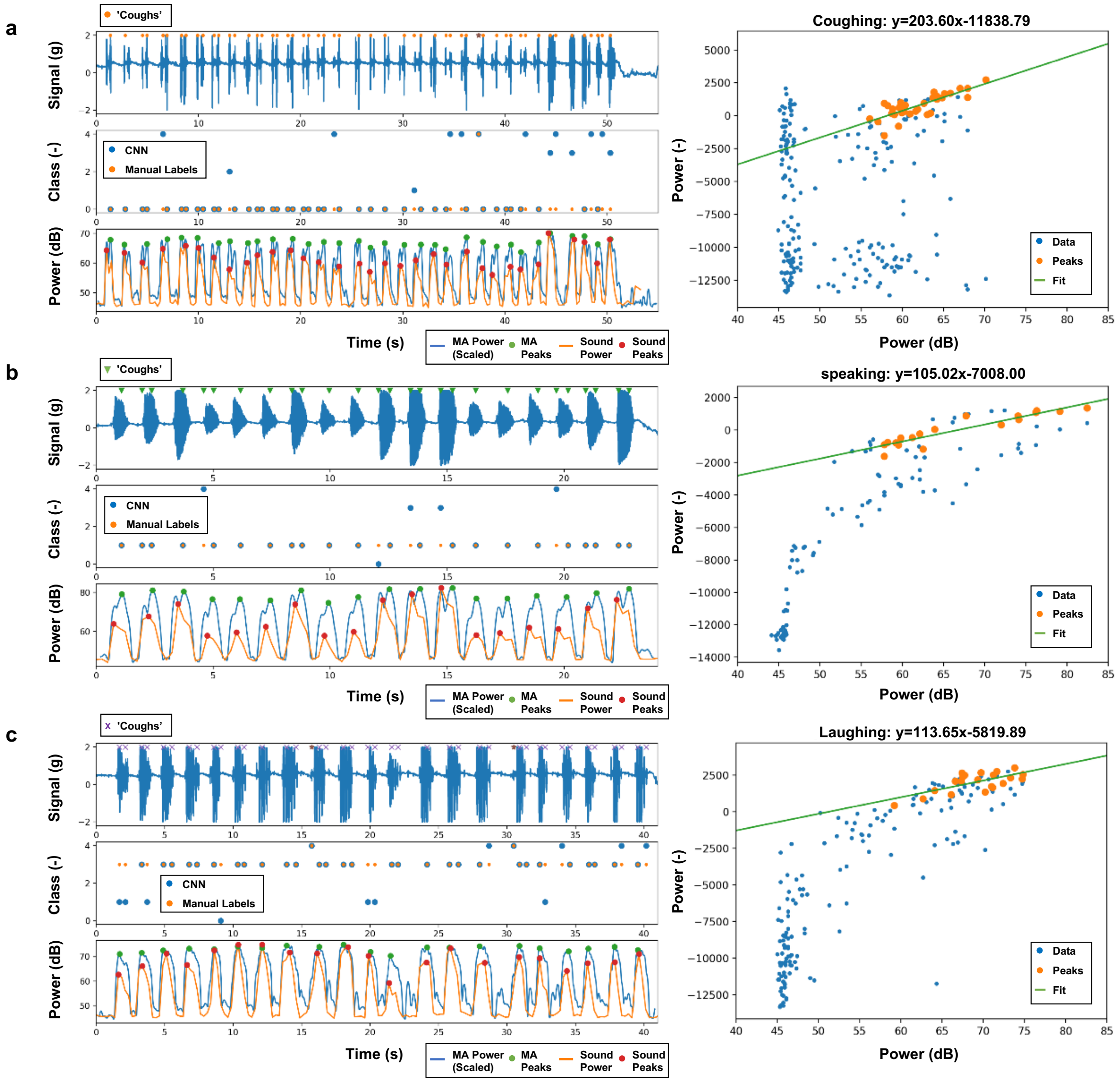

Fig. S1. Mechano-acoustic measurement of vocal event intensity. The time series of MA measurement, CNN-classification of the detected cough-like events in comparison with the manual labeling, and the scaled MA power synced with the decibel meter measurement of sound power for (a) coughing, (b) speaking, and (c) Laughing. Figures on the right show the correlation between MA power and sound power.

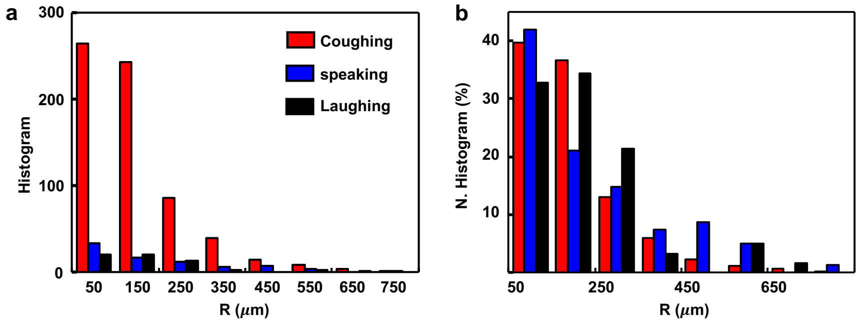

Fig. S2. Histogram of droplet sizes for coughing, speaking and laughing events. (a) Unnormalized and (b) Normalized histograms.

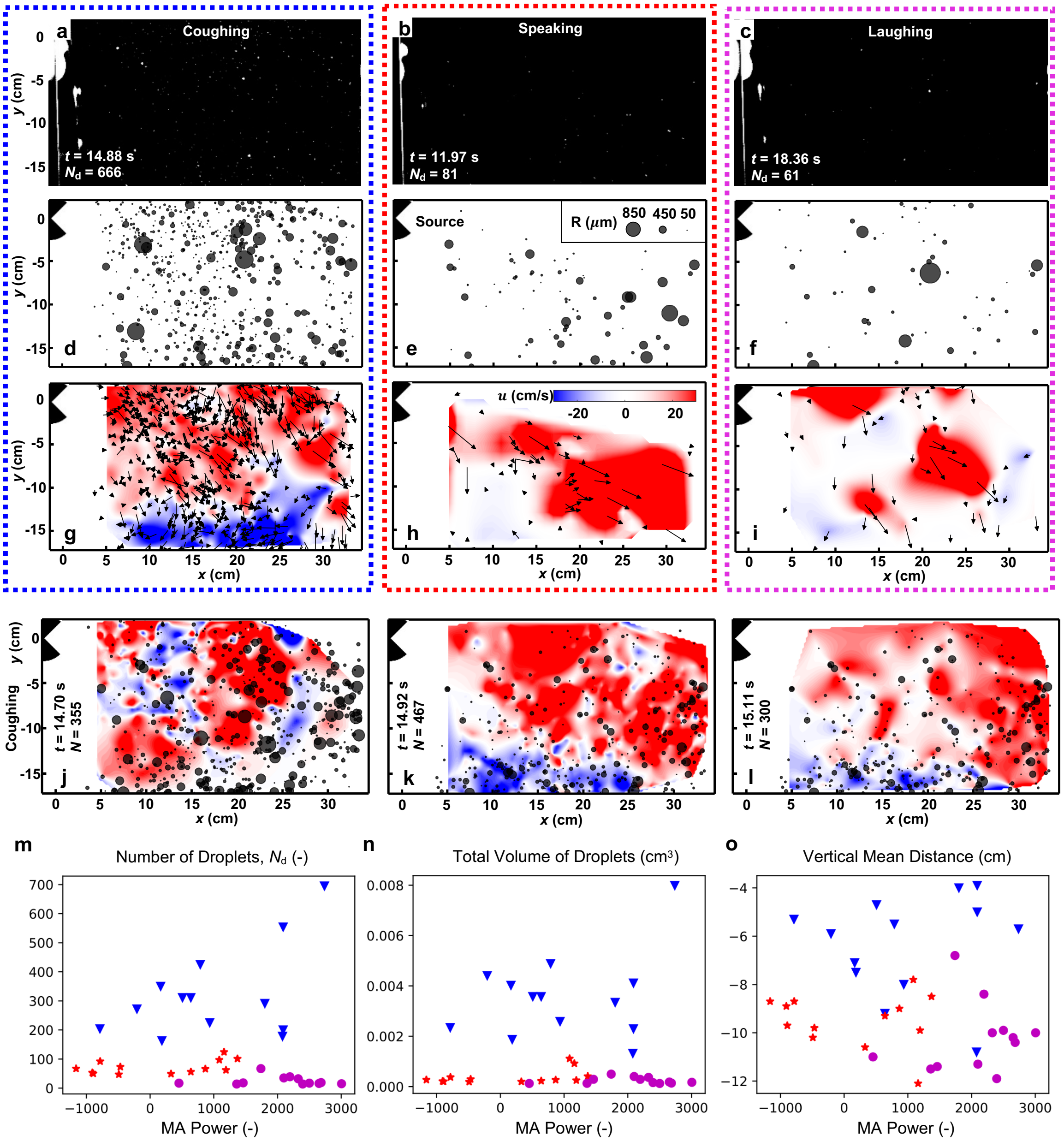

Fig. S3. Additional data on droplet dynamics. (a,b,c) Images of coughing, speaking, and laughing events at representative instants. (d,e,f) Detected droplets with sizes indicated by the diameters of the grey circular symbols (g,h,i) velocity vector and contour fields at the times corresponding to those in (a,b,c); the color denotes streamwise velocity. (j,k,l) Sequence of coughing at t=14.70s,14.92s,15.11s. (m,n,o) The total number, total volume, and vertical mean distance of produced droplets vs. MA power for coughing (blue), speaking (red), and laughing (magenta).

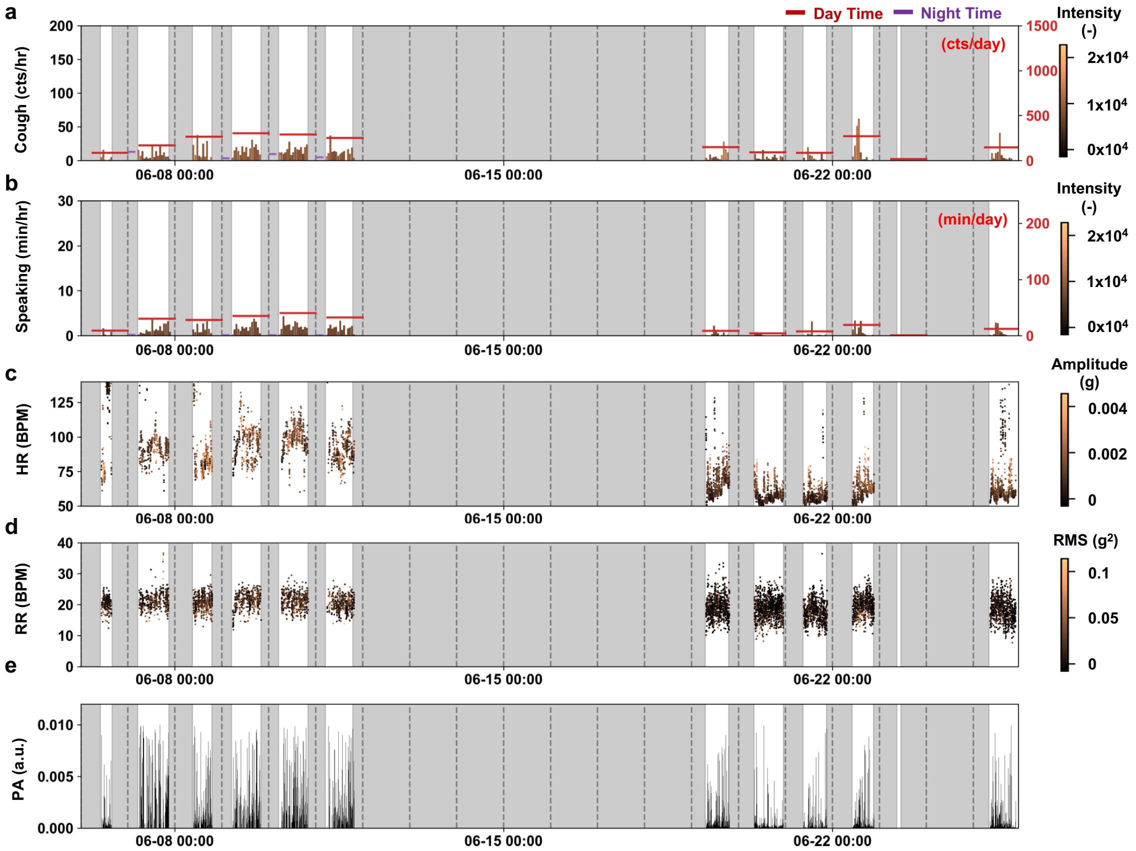

Fig. S4. Long-term monitoring of respiratory biomarkers and vital signs. Results for (a) coughing, (b) speaking, (c) heart rate, (d) respiration rate, and (e) physical activity of COVID-19 patient # SRALRN5F.

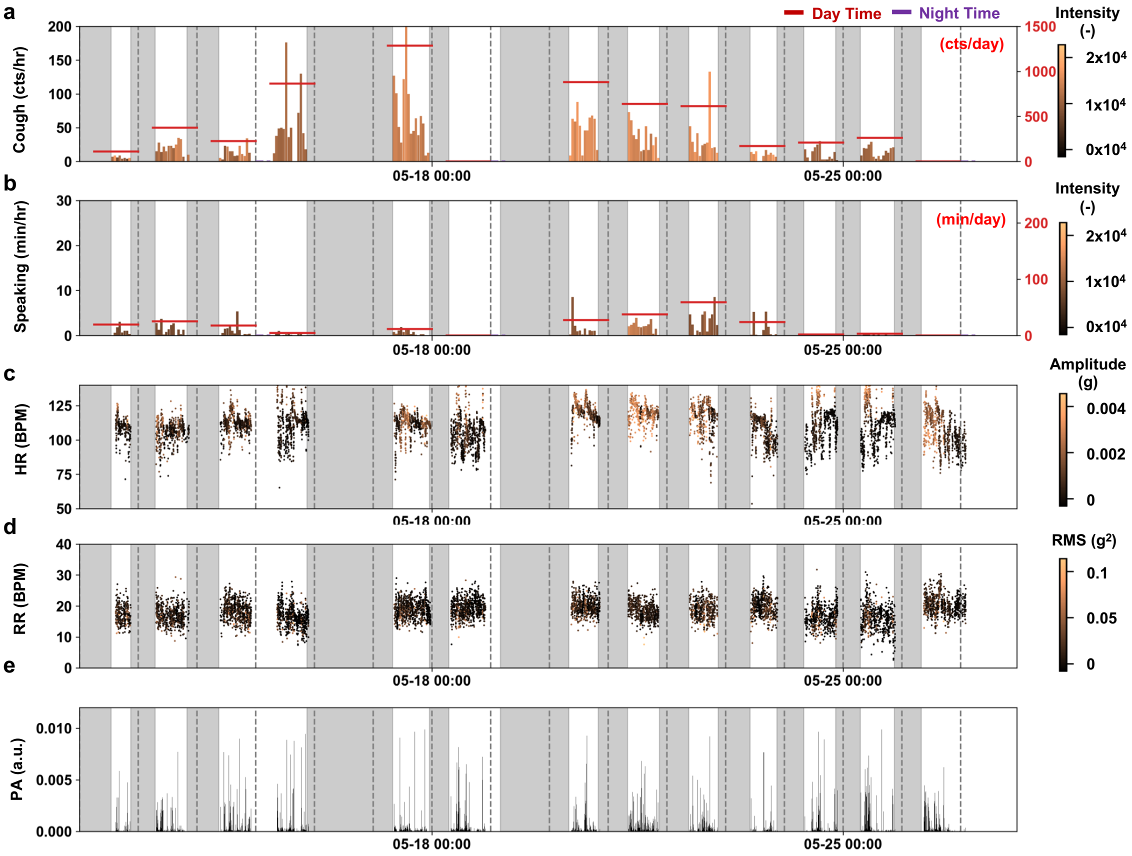

Fig. S5. Long-term monitoring of respiratory biomarkers and vital signs. Results for (a) coughing, (b) speaking, (c) heart rate, (d) respiration rate, and (e) physical activity of COVID-19 patient # SRAL2014F.

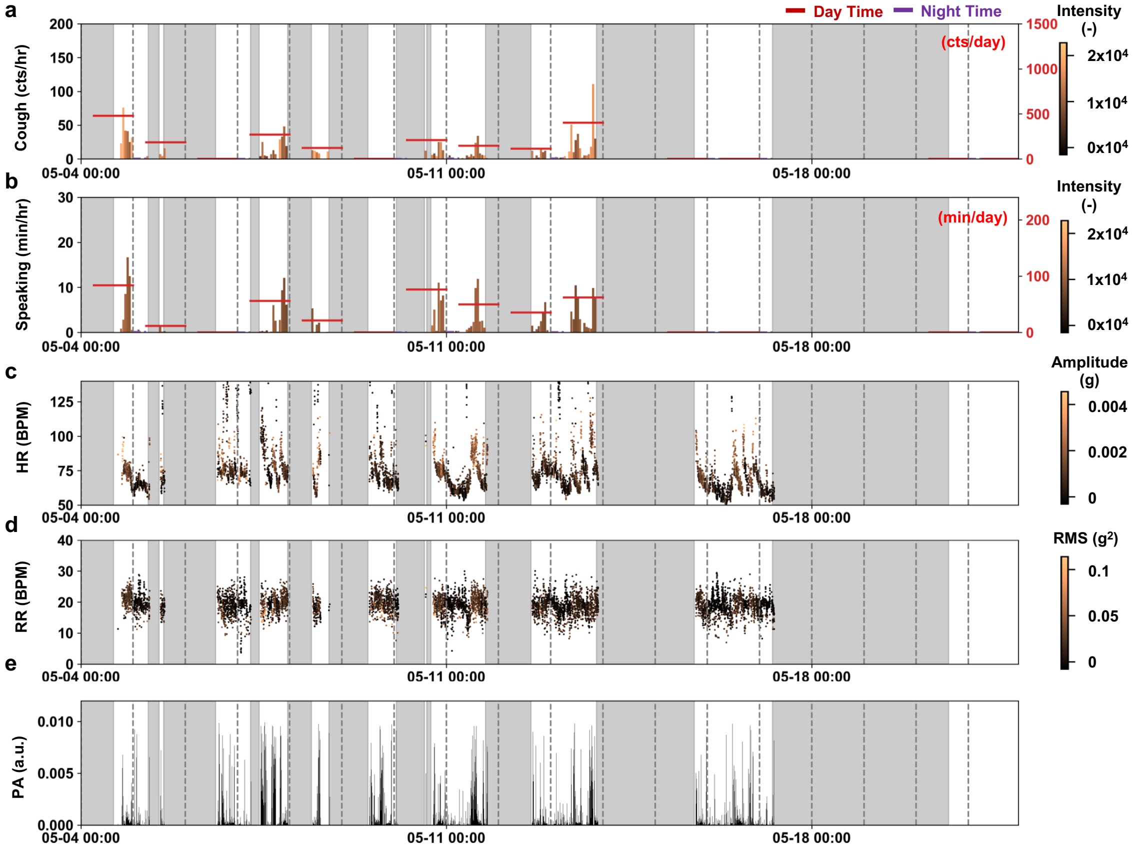

Fig. S6. Long-term monitoring of respiratory biomarkers and vital signs. Results for (a) coughing, (b) speaking, (c) heart rate, (d) respiration rate, and (e) physical activity of COVID-19 patient # SRALH11F.

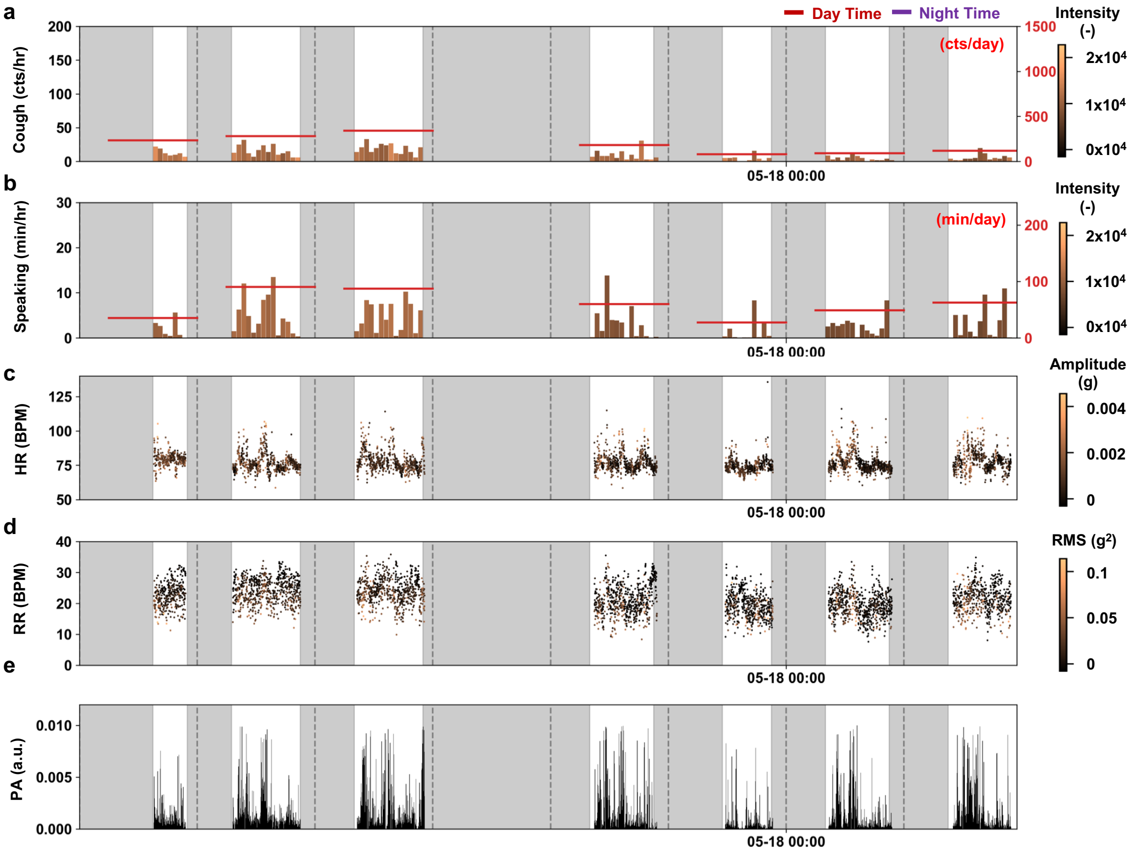

Fig. S7. Long-term monitoring of respiratory biomarkers and vital signs. Results for (a) coughing, (b) speaking, (c) heart rate, (d) respiration rate, and (e) physical activity of COVID-19 patient # SRAL2024M.

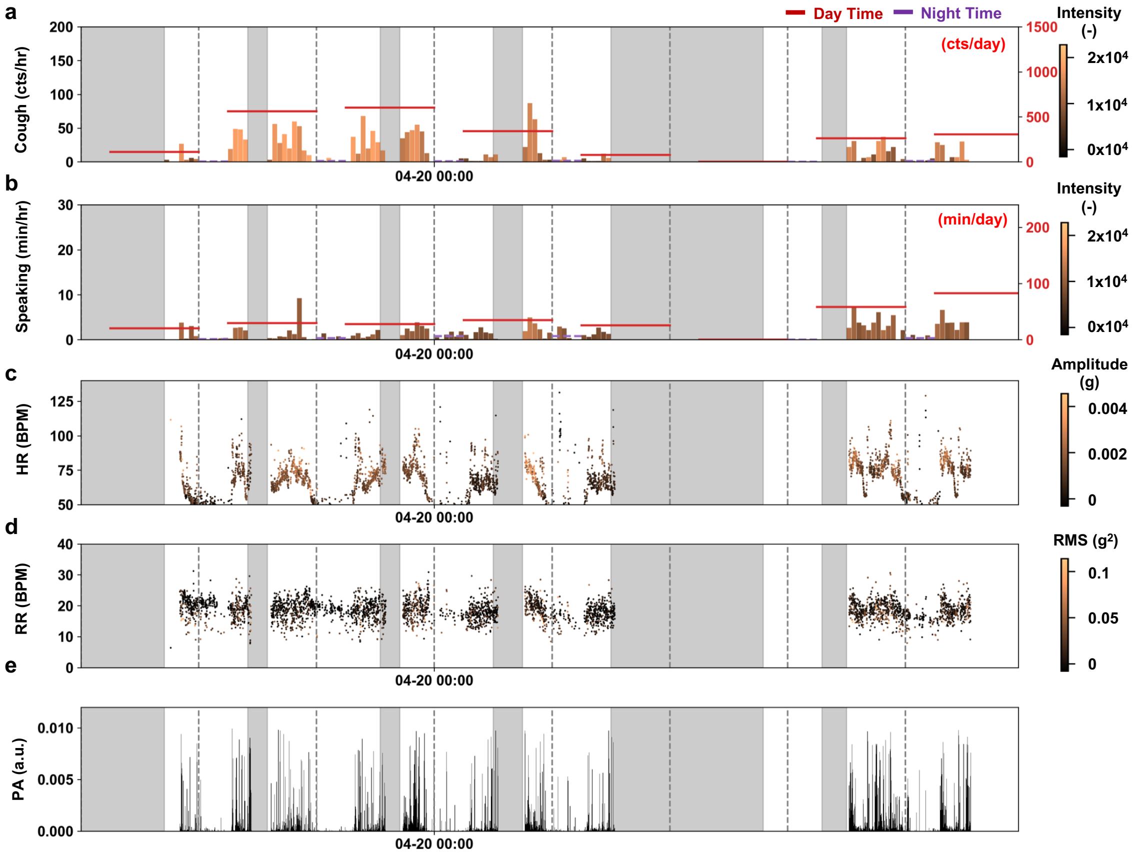

Fig. S8. Long-term monitoring of respiratory biomarkers and vital signs. Results for (a) coughing, (b) speaking, (c) heart rate, (d) respiration rate, and (e) physical activity of COVID-19 patient # SRAL-M-H2.

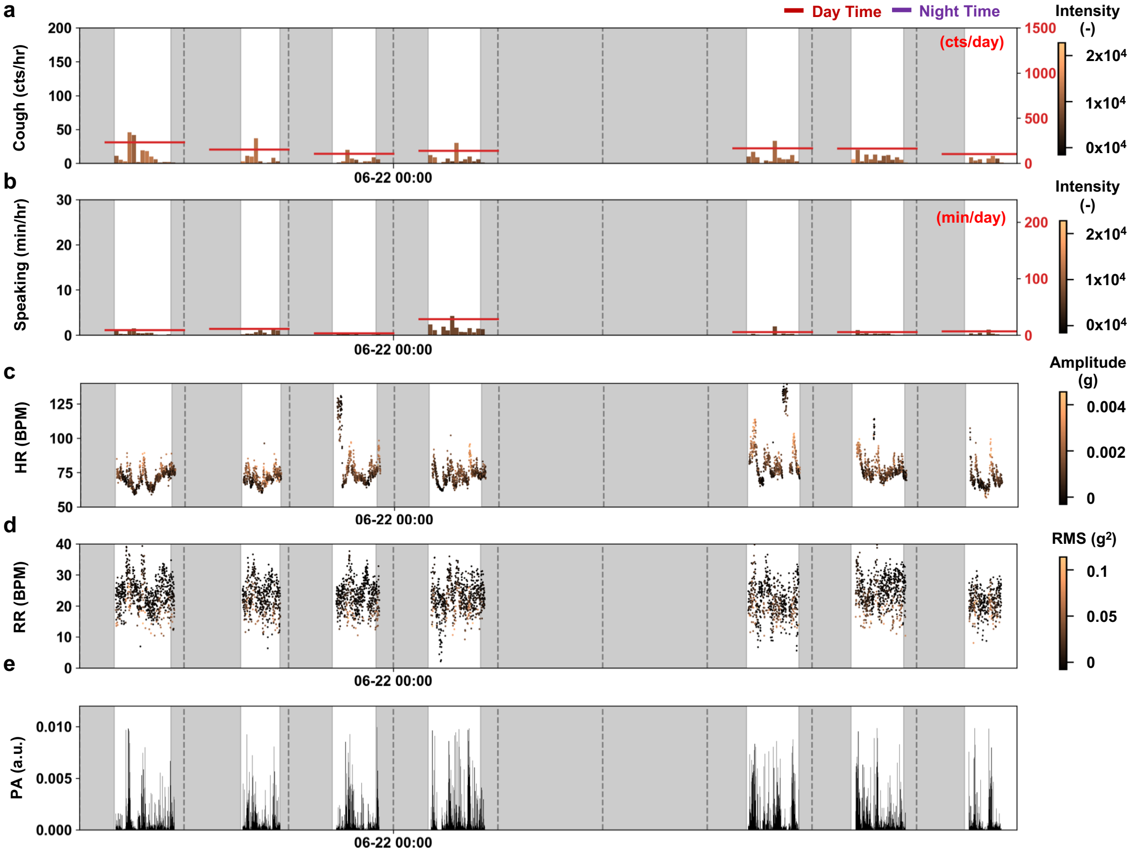

Fig. S9. Long-term monitoring of respiratory biomarkers and vital signs. Results for (a) coughing, (b) speaking, (c) heart rate, (d) respiration rate, and (e) physical activity of COVID-19 patient # SRAL2012BM.

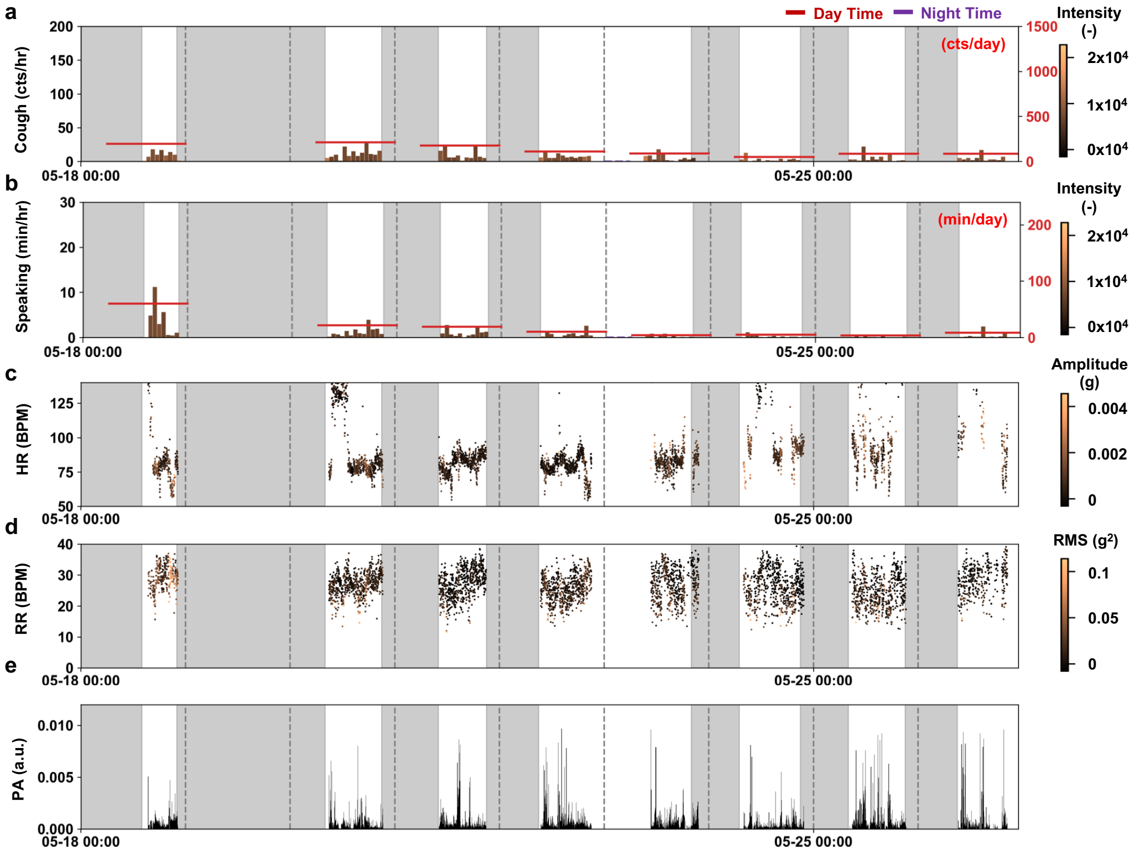

Fig. S10. Long-term monitoring of respiratory biomarkers and vital signs. Results for (a) coughing, (b) speaking, (c) heart rate, (d) respiration rate, and (e) physical activity of COVID-19 patient # SRAL2021F.

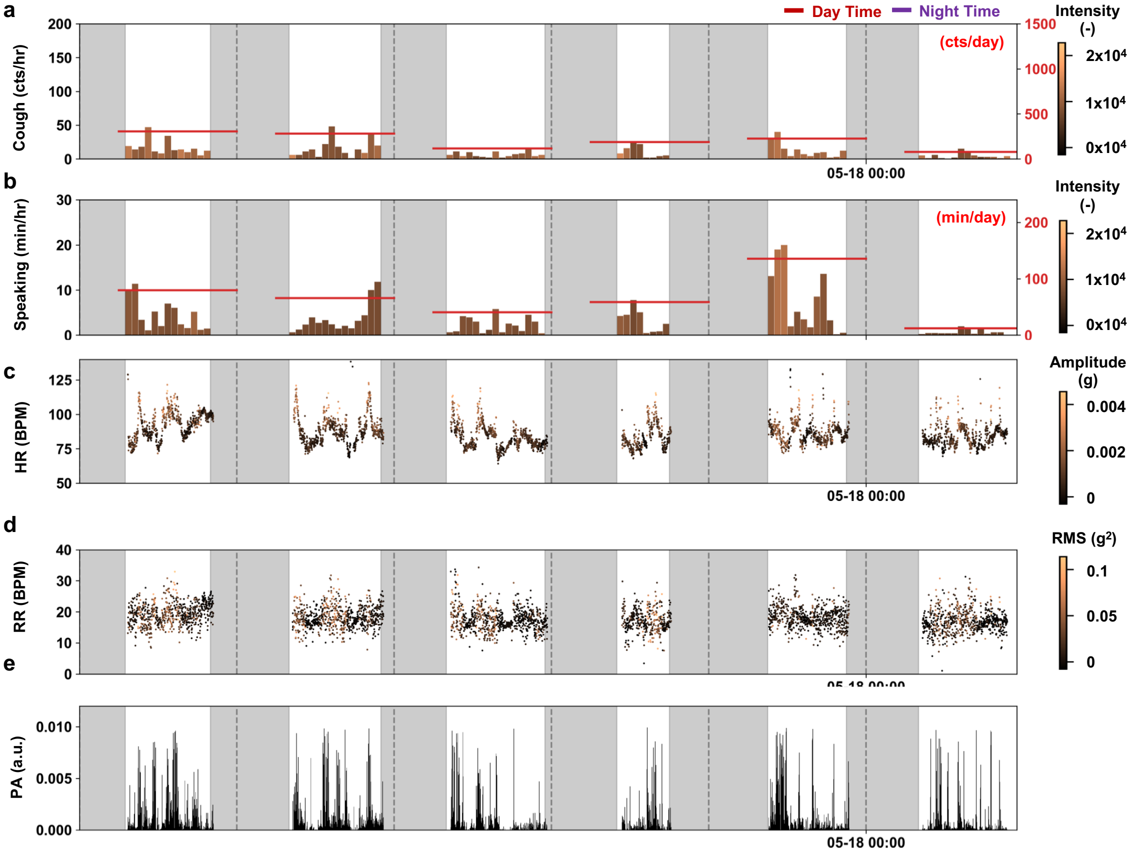

Fig. S11. Long-term monitoring of respiratory biomarkers and vital signs. Results for (a) coughing, (b) speaking, (c) heart rate, (d) respiration rate, and (e) physical activity of COVID-19 patient # SRAL2032F.

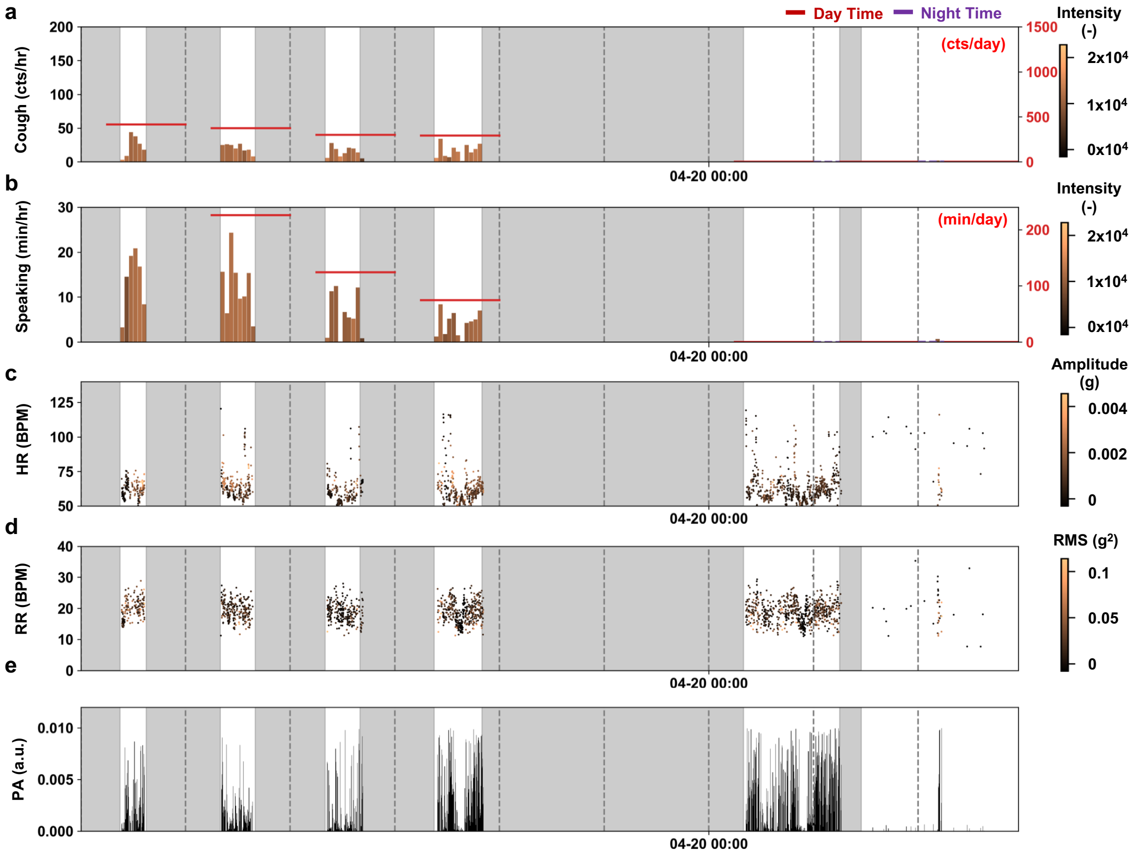

Fig. S12. Long-term monitoring of respiratory biomarkers and vital signs. Results for (a) coughing, (b) speaking, (c) heart rate, (d) respiration rate, and (e) physical activity of COVID-19 patient # SRAL7-M-MGR.

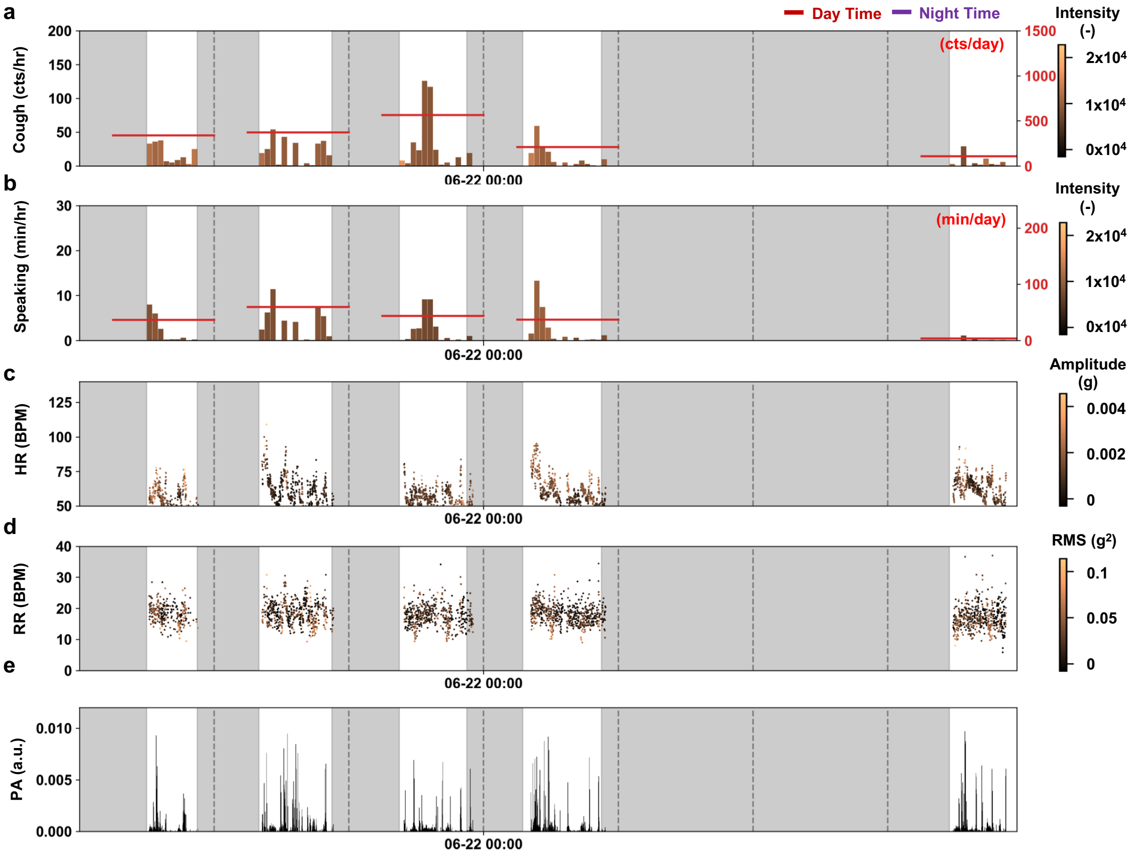

Fig. S13. Long-term monitoring of respiratory biomarkers and vital signs. Results for (a) coughing, (b) speaking, (c) heart rate, (d) respiration rate, and (e) physical activity of COVID-19 patient # SRAL1921F.

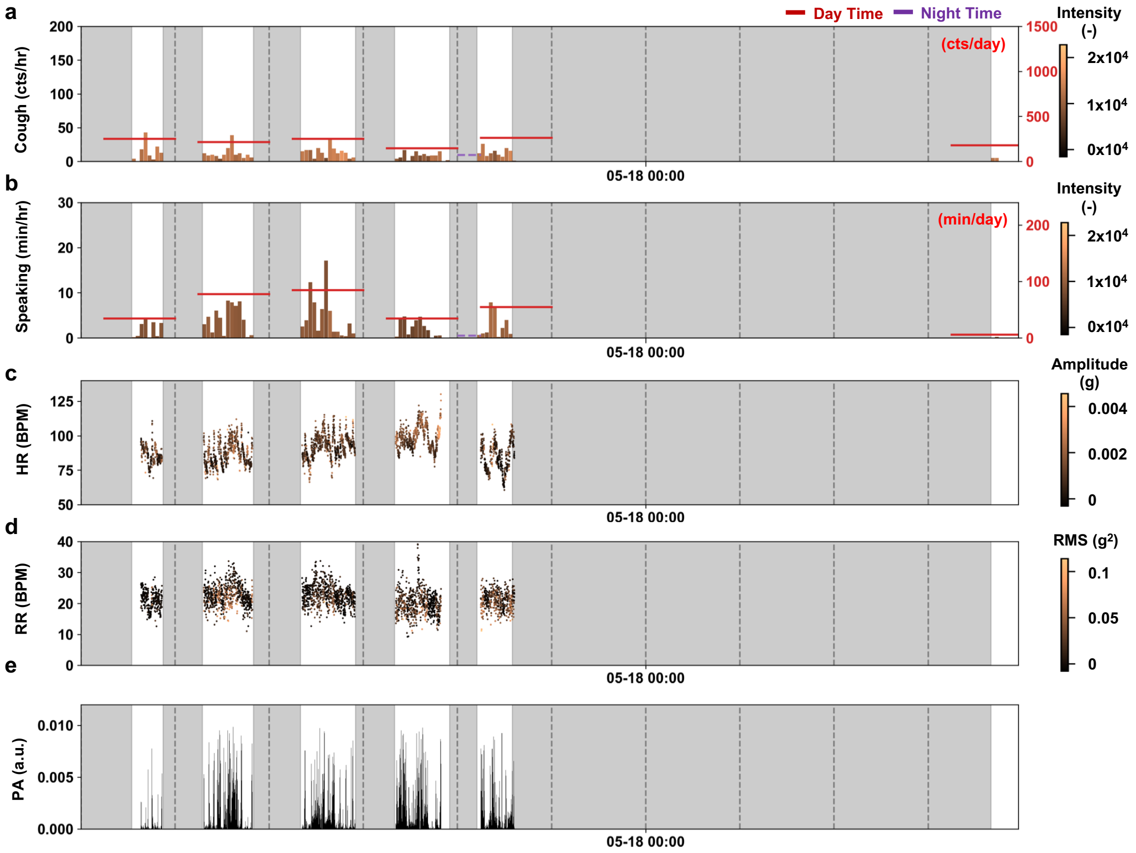

Fig. S14. Long-term monitoring of respiratory biomarkers and vital signs. Results for (a) coughing, (b) speaking, (c) heart rate, (d) respiration rate, and (e) physical activity of COVID-19 patient # SRAL2031M.

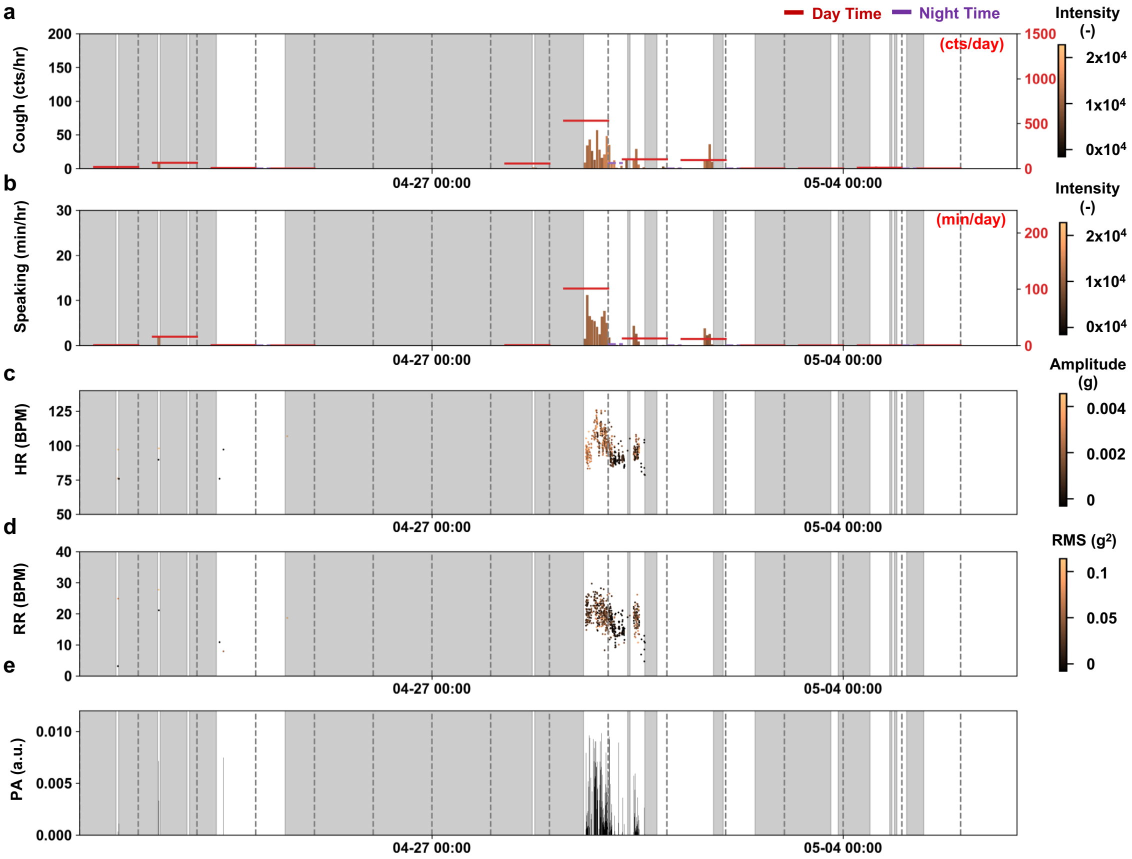

Fig. S15. Long-term monitoring of respiratory biomarkers and vital signs. Results for (a) coughing, (b) speaking, (c) heart rate, (d) respiration rate, and (e) physical activity of COVID-19 patient # NM12F.

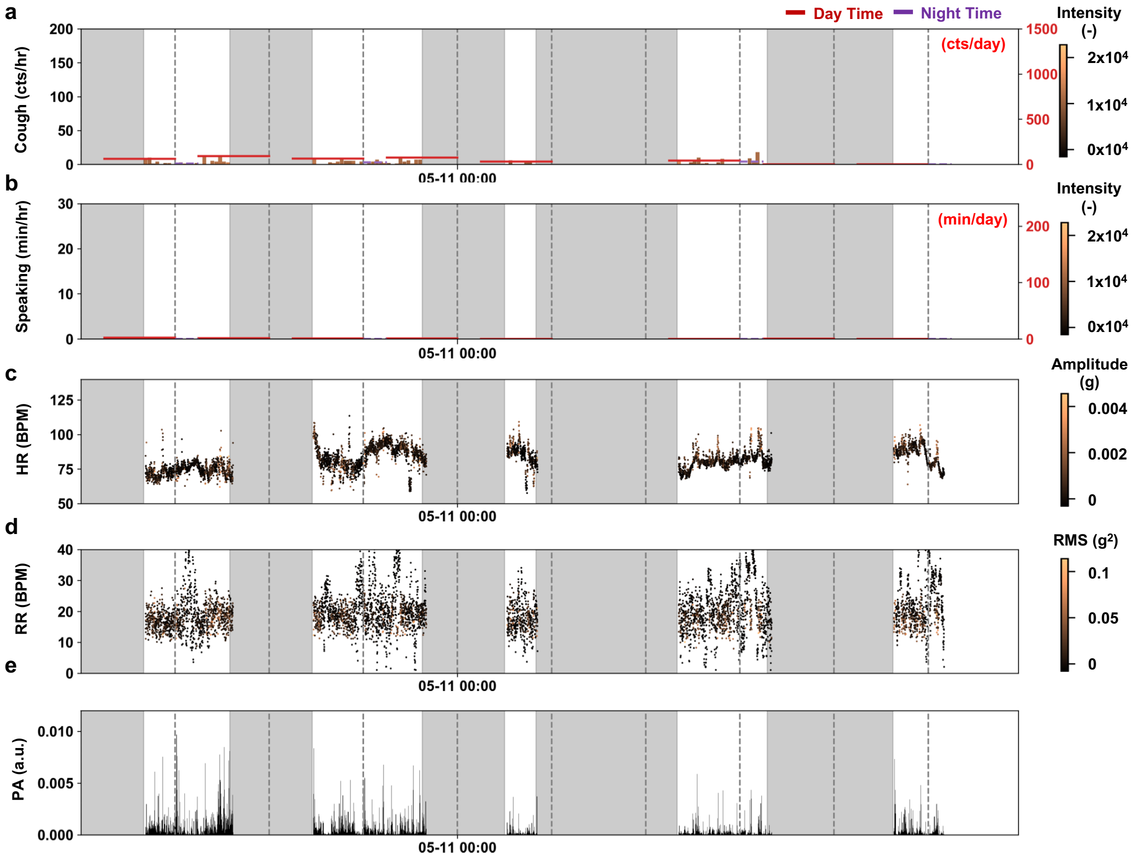

Fig. S16. Long-term monitoring of respiratory biomarkers and vital signs. Results for (a) coughing, (b) speaking, (c) heart rate, (d) respiration rate, and (e) physical activity of COVID-19 patient # NM17m.

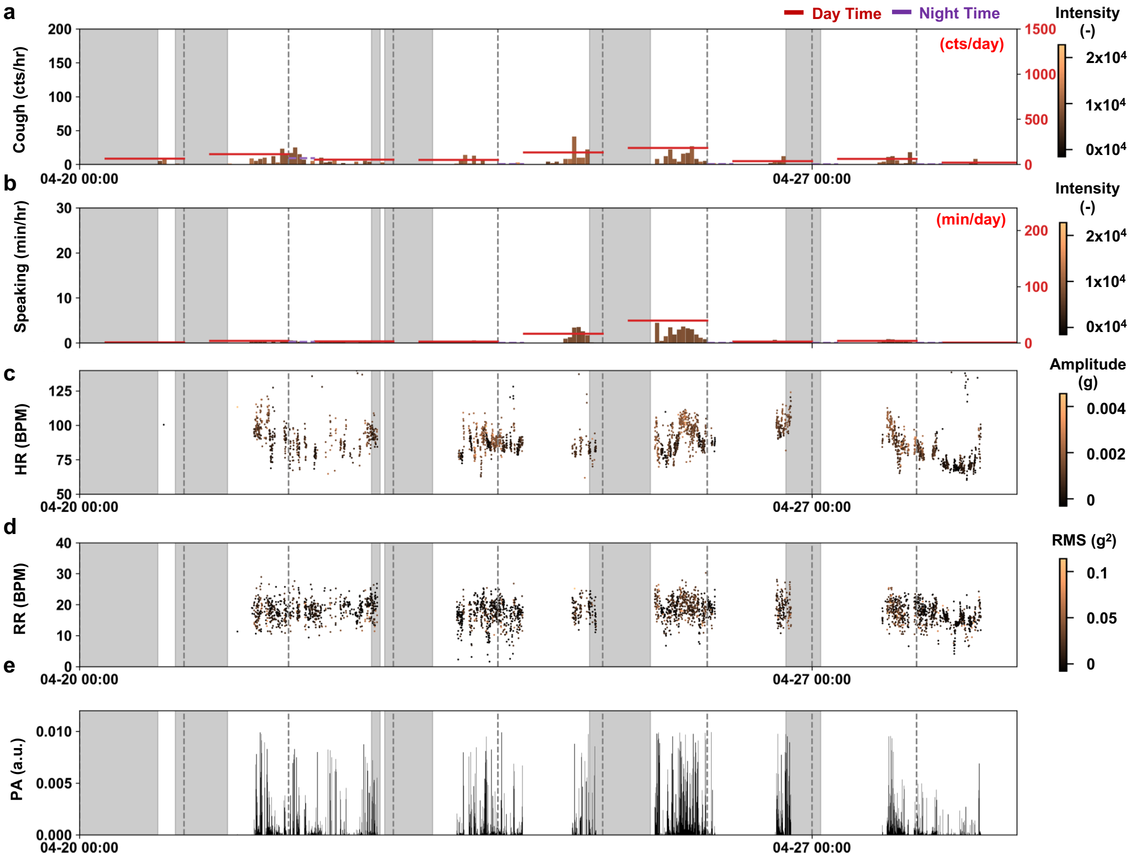

Fig. S17. Long-term monitoring of respiratory biomarkers and vital signs. Results for (a) coughing, (b) speaking, (c) heart rate, (d) respiration rate, and (e) physical activity of COVID-19 patient # SRAL-F-H5.

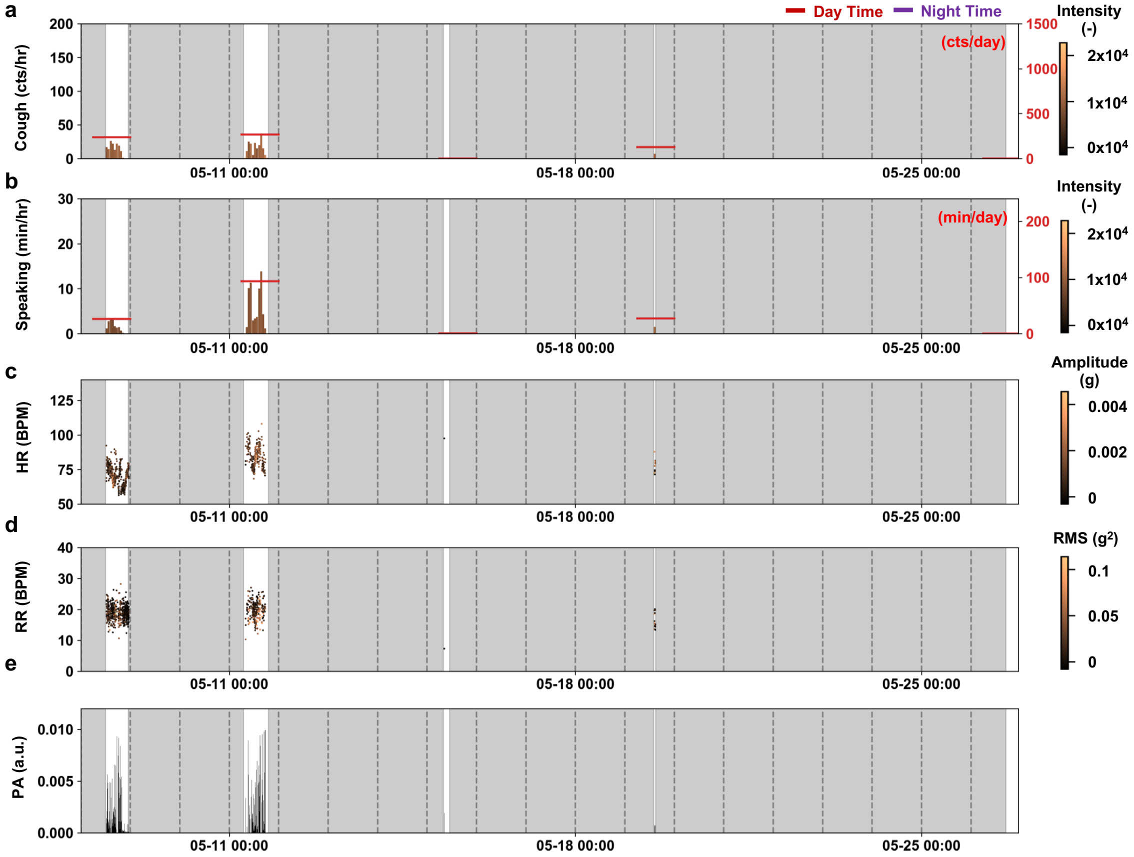

Fig. S18. Long-term monitoring of respiratory biomarkers and vital signs. Results for (a) coughing, (b) speaking, (c) heart rate, (d) respiration rate, and (e) physical activity of COVID-19 patient # SRALPT1F.

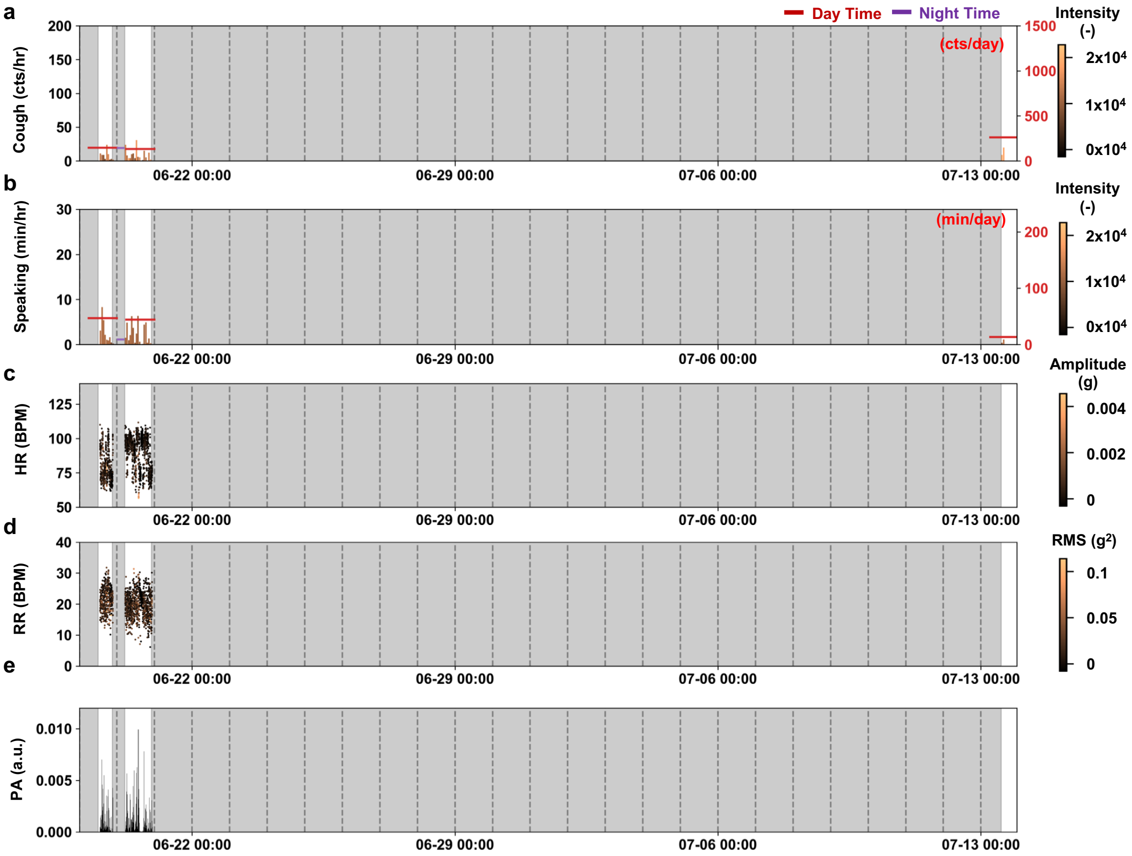

Fig. S19. Long-term monitoring of respiratory biomarkers and vital signs. Results for (a) coughing, (b) speaking, (c) heart rate, (d) respiration rate, and (e) physical activity of COVID-19 patient # SRAL1923M.

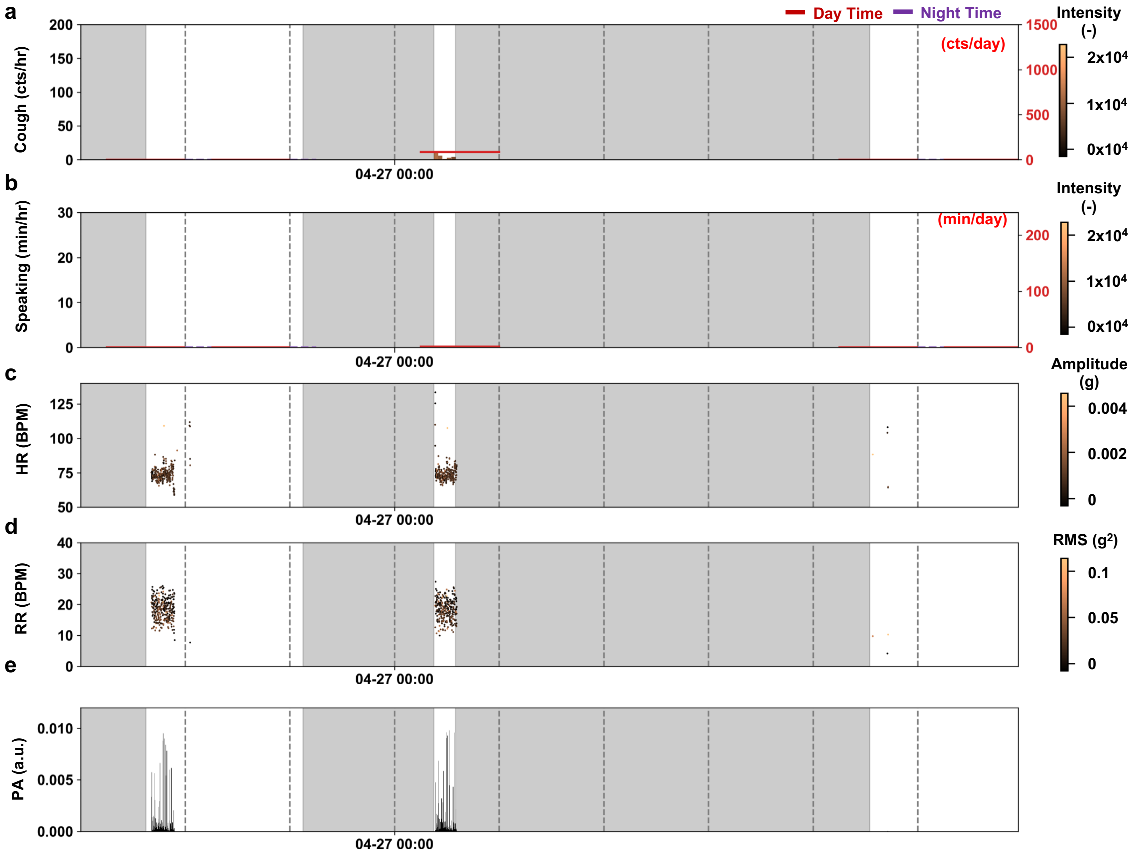

Fig. S20. Long-term monitoring of respiratory biomarkers and vital signs. Results for (a) coughing, (b) speaking, (c) heart rate, (d) respiration rate, and (e) physical activity of COVID-19 patient # SRALH8M.

Table S1. Demographic information for all the participants in the study.

Usage	#	ID	Gender	Age	Status	Start Date	Days of Enrollment	Hours of recording	PCR+ Date
Testing	1	SRAL-F-H5	F	25	Patient	20-04-20	8	143	NR
Testing	2	SRALPT1F	F	30	Patient	20-05-08	19	29	NR
Testing	3	NM12F	F	45	Patient	20-04-21	15	114	20-04-18
Testing	4	SRALH11F	F	52	Patient	20-05-04	17	182	20-04-09
Testing	5	SRALDOC3F	F	52	Patient	20-04-23	5	16	NR
Testing	6	NM15F	F	53	Patient	20-04-24	31	135	20-04-03
Testing	7	SRAL2032F	F	59	Patient	20-05-13	7	77	NR
Testing	8	SRAL2030F	F	65	Patient	20-05-12	3	35	NR
Testing	9	SRAL2014F	F	65	Patient	20-05-12	15	188	20-05-03
Testing	10	SRAL2023F	F	66	Patient	20-05-20	3	42	NR
Testing	11	SRAL2022F	F	69	Patient	20-05-13	6	56	NR
Testing	12	SRAL2021F	F	72	Patient	20-05-18	9	107	NR
Testing	13	SRALRN3F	F	NR	Patient	20-05-08	6	92	NR
Testing	14	SRAL1921F	F	NR	Patient	20-06-19	7	58	NR
Testing	15	SRALRN5F	F	NR	Patient	20-06-06	20	142	NR
Testing	16	SRAL2033M	M	32	Patient	20-05-22	6	59	NR
Testing	17	SRAL-M-H2	M	34	Patient	20-04-17	7	113	20-04-11
Testing	18	SRAL7-M-MGR	M	41	Patient	20-04-14	8	88	NR
Testing	19	SRALH8M	M	45	Patient	20-04-24	8	74	NR
Testing	20	SRAL2015M	M	52	Patient	20-05-12	4	44	NR
Testing	21	SRAL2024BM	M	55	Patient	20-05-27	6	51	NR
Testing	22	SRAL2012BM	M	55	Patient	20-06-19	9	77	NR
Testing	23	NM17M	M	56	Patient	20-05-07	9	114	20-05-05
Testing	24	SRAL2024M	M	60	Patient	20-05-12	8	83	20-03-28
Testing	25	SRAL2031M	M	61	Patient	20-05-12	10	60	20-04-21
Testing	26	SRAL1922M	M	NR	Patient	20-06-19	1	6	NR
Testing	27	SRAL1923M	M	NR	Patient	20-06-19	25	31	NR
Training	1	NU-M-1	M	37	Healthy	20-05-09	1	5	NA
Training	2	NU-M-2	M	53	Healthy	20-05-02	1	5	NA
Training	3	NU-M-3	M	29	Healthy	20-05-09	1	6	NA
Training	4	NU-F-1	F	26	Healthy	20-05-13	1	8	NA
Training	5	NU-M-4	M	30	Healthy	20-05-11	1	24	NA
Training	6	NU-F-2	F	29	Healthy	20-05-13	1	23	NA
Training	7	NU-F-3	F	29	Healthy	20-05-15	1	22	NA
Training	8	NU-M-5	M	40	Healthy	20-05-15	1	18	NA
Training	9	NU-F-4	F	27	Healthy	20-05-16	2	19	NA
Training	10	NU-M-6	M	25	Healthy	20-06-10	1	2	NA
Training	11	SRAL-F-H1	F	31	Patient	20-04-17	10	115	NR
Training	12	SRAL-F-H4	F	21	Patient	20-04-21	9	142	NR
Training	13	SRAL-F-H6	F	48	Patient	20-04-21	9	113	NR
Training	14	SRAL-M-H3	M	59	Patient	20-04-17	13	171	NR
Training	15	SRAL2020BF	F	64	Patient	20-05-29	13	38	NR
Training	16	SRAL2025M	M	75	Patient	20-05-19	8	101	NR
Training	17	SRAL2026F	F	68	Patient	20-05-21	4	58	NR
Training	18	SRAL2032BM	M	52	Patient	20-05-29	7	60	NR
Training	19	SRAL2019M	M	60	Patient	20-06-05	10	38	NR
Training	20	SRAL2006M	M	59	Patient	20-06-19	8	59	NR
Note:									
NR = Not Reported									
NA=Not Applicable									

Table S2. Detailed statistical information of the performance of the CNN model on subjects.

Usage	#	ID	Cough				Speak				Throat clearing				Laugh				Motion artifact			
			Labeled # of events	sens.	spec.	prec.	Labeled # of events	sens.	spec.	prec.	Labeled # of events	sens.	spec.	prec.	Labeled # of events	sens.	spec.	prec.	Labeled # of events	sens.	spec.	prec.
Testing	1	SRAL-F-H5																				
Testing	2	SRALPT1F																				
Testing	3	NM12F																				
Testing	4	SRALH11F	185	0.98	0.86	0.71	182	0.64	1.00	1.00	62	0.92	0.97	0.74	95	0.54	0.97	0.73	183	0.97	0.98	0.94
Testing	5	SRALDOC3F																				
Testing	6	NM15F	292	0.97	0.98	0.95	291	0.96	1.00	1.00	51	0.98	1.00	1.00	51	0.80	0.99	0.84	291	1.00	0.99	0.97
Testing	7	SRAL2032F	384	0.76	0.96	0.88	381	0.47	0.98	0.90	119	0.89	0.89	0.43	145	0.84	0.91	0.50	382	0.88	0.95	0.86
Testing	8	SRAL2030F																				
Testing	9	SRAL2014F	406	0.78	0.98	0.95	404	0.74	0.98	0.96	62	0.94	0.94	0.44	13	0.92	0.94	0.14	404	0.91	0.94	0.87
Testing	10	SRAL2023F																				
Testing	11	SRAL2022F																				
Testing	12	SRAL2021F	410	0.69	0.98	0.93	407	0.78	0.99	0.96	184	0.97	0.90	0.59	11	0.73	1.00	0.57	408	1.00	0.94	0.86
Testing	13	SRALRN3F																				
Testing	14	SRAL1921F	232	1.00	0.95	0.87	229	0.93	1.00	1.00	99	1.00	1.00	1.00	117	0.69	0.98	0.86	232	1.00	0.99	0.98
Testing	15	SRALRN5F																				
Testing	16	SRAL2033M																				
Testing	17	SRAL-M-H2	301	0.99	1.00	0.99	298	0.97	1.00	1.00	101	0.98	1.00	0.99	86	0.99	0.99	0.86	300	0.99	1.00	1.00
Testing	18	SRAL7-M-MGR	180	0.71	0.89	0.62	177	0.47	0.89	0.52	179	0.55	0.92	0.64	178	0.51	0.92	0.62	177	0.75	0.87	0.59
Testing	19	SRALH8M																				
Testing	20	SRAL2015M																				
Testing	21	SRAL2024BM																				
Testing	22	SRAL2012BM	287	0.94	0.97	0.92	284	0.64	0.98	0.93	249	0.90	0.94	0.82	12	0.92	0.96	0.19	285	0.93	0.96	0.90
Testing	23	NM17M																				
Testing	24	SRAL2024M	108	0.86	0.88	0.69	105	0.50	0.99	0.91	106	0.77	0.91	0.73	27	0.93	0.94	0.51	106	0.88	0.99	0.95
Testing	25	SRAL2031M																				
Testing	26	SRAL1922M																				
Testing	27	SRAL1923M																				
Training	1	NU-M-1	126	1.00	0.99	0.97	221	0.98	0.99	0.97	124	0.88	0.99	0.96	146	0.99	0.99	0.95	157	0.99	1.00	1.00
Training	2	NU-M-2	184	0.96	0.98	0.90	123	0.97	0.96	0.77	179	0.74	0.99	0.96	206	0.88	0.98	0.91	304	1.00	0.99	0.98
Training	3	NU-M-3	143	1.00	0.99	0.92	291	0.89	0.96	0.90	133	0.92	0.98	0.87	177	0.82	0.99	0.92	277	0.99	1.00	0.99
Training	4	NU-F-1	104	0.95	1.00	1.00	117	0.99	0.89	0.65	96	0.51	0.99	0.89	77	0.78	1.00	1.00	309	0.99	0.99	0.99
Training	5	NU-M-4	120	0.78	0.99	0.96	89	0.63	1.00	0.97	215	0.94	0.98	0.95	28	0.89	0.96	0.47	321	1.00	0.93	0.91
Training	6	NU-F-2	128	0.98	0.99	0.95	0	nan	0.99	0.29	155	0.98	0.99	0.97	103	0.87	1.00	1.00	315	1.00	0.99	0.99
Training	7	NU-F-3	98	0.87	1.00	0.99	179	0.92	0.97	0.89	0	nan	0.98	0.00	214	0.87	0.97	0.92	289	1.00	1.00	1.00
Training	8	NU-M-5	146	0.97	0.97	0.91	28	0.71	0.96	0.45	169	0.76	0.98	0.93	32	0.91	0.98	0.73	301	0.99	0.99	0.99
Training	9	NU-F-4	217	0.98	0.98	0.93	236	0.88	0.96	0.86	211	0.96	0.98	0.90	224	0.78	0.99	0.94	293	1.00	0.99	0.98
Training	10	NU-M-6	113	0.92	0.94	0.70	157	1.00	0.96	0.85	141	0.64	1.00	0.99	106	0.77	0.99	0.92	324	1.00	0.99	0.99
Training	11	SRAL-F-H1	166	0.85	0.99	0.97	169	0.88	0.98	0.94	39	0.67	0.97	0.58	29	0.76	0.97	0.58	250	0.99	0.95	0.93
Training	12	SRAL-F-H4	114	0.86	0.92	0.70	42	0.81	0.98	0.69	0	nan	0.98	0.00	87	0.36	1.00	1.00	416	0.97	0.90	0.94
Training	13	SRAL-F-H6	114	0.88	0.98	0.89	166	0.84	1.00	1.00	0	nan	0.99	0.00	0	nan	0.98	0.00	443	0.97	0.93	0.96
Training	14	SRAL-M-H3	47	0.91	0.99	0.91	200	0.92	0.98	0.92	55	0.69	0.99	0.79	47	0.81	0.99	0.81	495	0.99	0.96	0.97
Training	15	SRAL2020BF	165	0.90	1.00	0.99	102	0.95	0.99	0.95	0	nan	0.99	0.00	0	nan	0.99	0.00	179	1.00	0.97	0.96
Training	16	SRAL2025M	163	0.91	0.99	0.97	121	0.88	0.99	0.94	116	0.80	0.99	0.97	0	nan	0.97	0.00	190	0.97	0.94	0.89
Training	17	SRAL2026F	43	0.70	1.00	1.00	0	nan	0.99	0.00	0	nan	0.97	0.00	0	nan	0.97	0.00	30	1.00	0.81	0.79
Training	18	SRAL2032BM	24	0.79	0.98	0.59	199	0.84	0.96	0.93	0	nan	0.94	0.00	30	0.80	0.96	0.56	310	0.86	0.98	0.98
Training	19	SRAL2019M	314	0.90	0.96	0.92	377	0.80	0.98	0.96	0	nan	0.98	0.00	0	nan	0.94	0.00	282	0.90	0.98	0.94
Training	20	SRAL2006M	255	0.85	0.95	0.92	73	0.85	0.98	0.83	0	nan	0.99	0.00	0	nan	0.99	0.00	310	0.94	0.92	0.91

Movie M1. Videos of raw and detected droplets for representative talking, laughing and coughing events. The videos play 10 times slower than the actual speed.
